# Comparative genomics of the major fungal agents of human and animal Sporotrichosis: *Sporothrix schenckii* and *Sporothrix brasiliensis*

**DOI:** 10.1186/1471-2164-15-943

**Published:** 2014-10-29

**Authors:** Marcus M Teixeira, Luiz GP de Almeida, Paula Kubitschek-Barreira, Fernanda L Alves, Érika S Kioshima, Ana KR Abadio, Larissa Fernandes, Lorena S Derengowski, Karen S Ferreira, Rangel C Souza, Jeronimo C Ruiz, Nathalia C de Andrade, Hugo C Paes, André M Nicola, Patrícia Albuquerque, Alexandra L Gerber, Vicente P Martins, Luisa DF Peconick, Alan Viggiano Neto, Claudia B Chaucanez, Patrícia A Silva, Oberdan L Cunha, Fabiana FM de Oliveira, Tayná C dos Santos, Amanda LN Barros, Marco A Soares, Luciana M de Oliveira, Marjorie M Marini, Héctor Villalobos-Duno, Marcel ML Cunha, Sybren de Hoog, José F da Silveira, Bernard Henrissat, Gustavo A Niño-Vega, Patrícia S Cisalpino, Héctor M Mora-Montes, Sandro R Almeida, Jason E Stajich, Leila M Lopes-Bezerra, Ana TR Vasconcelos, Maria SS Felipe

**Affiliations:** Departamento de Biologia Celular, Universidade de Brasília, Brasília, DF Brazil; Laboratório Nacional de Computação Científica, Petrópolis, RJ Brazil; Departamento de Biologia Celular, Instituto de Biologia Roberto Alcântara Gomes, Universidade do Estado do Rio de Janeiro, Rio de Janeiro, RJ Brazil; Departamento de Microbiologia, Universidade Federal de Minas Gerais, Belo Horizonte, MG Brazil; Grupo Informática de Biossistemas, Centro de Pesquisas René Rachou, FIOCRUZ, Minas, Belo Horizonte, MG Brazil; Departamento de Análises Clínicas, Universidade Estadual de Maringá, Maringá, PR Brazil; Programa de Pós-Graduação em Ciências e Tecnologias em Saúde, Universidade de Brasília, Ceilândia, Brasília, DF Brazil; Instituto de Ciências Ambientais, Químicas e Farmacêuticas, Universidade Federal de São Paulo, Campus Diadema, São Paulo, SP Brazil; Pós-Graduação em Ciências Genômicas e Biotecnologia, Universidade Católica de Brasília, Brasília, DF Brazil; Programa de pós-graduação em Medicina Tropical, Universidade de Brasília, Brasília, DF Brazil; Programa de pós-graduação em Bioinformática, Universidade Federal de Minas Gerais, Minas Gerais, Brazil; Departamento de Microbiologia Imunobiologia e Parasitologia, Universidade Federal de São Paulo, São Paulo, SP Brazil; Centro de Microbiología y Biología Celular, Instituto Venezolano de Investigaciones Cientificas, Caracas, Venezuela; CBS-KNAW Fungal Biodiversity Centre, Utrecht, The Netherlands; Centre National de la Recherche Scientifique, Aix-Marseille, Université, CNRS, Marseille, France; Departamento de Biología, Universidad de Guanajuato, Guanajuato, Mexico; Departamento de Análises Clínicas e Toxicológicas, Universidade de São Paulo, São Paulo, SP Brazil; Department of Plant Pathology & Microbiology, University of California, Riverside, CA USA

**Keywords:** *Sporothrix schenckii*, *Sporothrix brasiliensis*, Comparative genomics, Fungal evolution

## Abstract

**Background:**

The fungal genus *Sporothrix* includes at least four human pathogenic species. One of these species, *S. brasiliensis*, is the causal agent of a major ongoing zoonotic outbreak of sporotrichosis in Brazil. Elsewhere, sapronoses are caused by *S. schenckii* and *S. globosa.* The major aims on this comparative genomic study are: 1) to explore the presence of virulence factors in *S. schenckii and S. brasiliensis*; 2) to compare *S. brasiliensis,* which is cat-transmitted and infects both humans and cats with *S. schenckii*, mainly a human pathogen; 3) to compare these two species to other human pathogens (Onygenales) with similar thermo-dimorphic behavior and to other plant-associated Sordariomycetes.

**Results:**

The genomes of *S. schenckii and S. brasiliensis* were pyrosequenced to 17x and 20x coverage comprising a total of 32.3 Mb and 33.2 Mb, respectively. Pair-wise genome alignments revealed that the two species are highly syntenic showing 97.5% average sequence identity. Phylogenomic analysis reveals that both species diverged about 3.8-4.9 MYA suggesting a recent event of speciation. Transposable elements comprise respectively 0.34% and 0.62% of the *S. schenckii* and *S. brasiliensis* genomes and expansions of *Gypsy*-like elements was observed reflecting the accumulation of repetitive elements in the *S. brasiliensis* genome. Mitochondrial genomic comparisons showed the presence of group-I intron encoding homing endonucleases (HE’s) exclusively in *S. brasiliensis*. Analysis of protein family expansions and contractions in the *Sporothrix* lineage revealed expansion of LysM domain-containing proteins, small GTPases, PKS type1 and leucin-rich proteins. In contrast, a lack of polysaccharide lyase genes that are associated with decay of plants was observed when compared to other Sordariomycetes and dimorphic fungal pathogens, suggesting evolutionary adaptations from a plant pathogenic or saprobic to an animal pathogenic life style.

**Conclusions:**

Comparative genomic data suggest a unique ecological shift in the *Sporothrix* lineage from plant-association to mammalian parasitism, which contributes to the understanding of how environmental interactions may shape fungal virulence. . Moreover, the striking differences found in comparison with other dimorphic fungi revealed that dimorphism in these close relatives of plant-associated Sordariomycetes is a case of convergent evolution, stressing the importance of this morphogenetic change in fungal pathogenesis.

**Electronic supplementary material:**

The online version of this article (doi:10.1186/1471-2164-15-943) contains supplementary material, which is available to authorized users.

## Background

The fungal genus *Sporothrix* includes about 60 species found on all inhabited continents mainly occurring as environmental saprobes, living in association with plants or decaying matter. One lineage within the genus is composed of at least four pathogenic species associated with human and animal sporotrichosis: *Sporothrix schenckii sensu stricto*, *S. brasiliensis*, *S. globosa*, and *S. luriei* [1–6]. Subcutaneous infections caused by *S. schenckii* are globally endemic [1, 2]. Additionally, outbreaks have been described from South Africa, Australia, China and India [7–10]. During the last two decades, an ongoing zoonotic outbreak of sporotrichosis has been observed in Brazil. Initially thought to be caused by *Sporothrix schenckii,* detailed studies demonstrated that most outbreak isolates were actually *S. brasiliensis* [6].

Sporothricosis is classically associated with rural activities such as agriculture, floriculture or hunting, but more recently felines have emerged as source of human infection. The most common clinical form is a chronic subcutaneous/lymphocutaneous disease acquired after inoculation of fungal material into the skin. Extracutaneous and disseminated forms secondary to cutaneous infection have been described in patients who are immunocompromised as a result of AIDS, chronic alcoholism and diabetes [11]. Rarely, severe cases involving pulmonary infection are noted [3, 4].

The pathogens of the genus *Sporothrix* exhibit a thermo-dimorphic phenotype: in its saprophytic stage or in *in vitro* culture at 25°C the fungus grows with its filamentous form characterized by hyaline, septate hyphae with sympodial conidiogenous cells that produce two types of spores: hyaline conidia that form clusters and brown, thick-walled spores that are distributed perpendicularly alongside the hyphae. During the parasitic stage, the fungus is found as cigar-shaped yeast cells that can also be obtained *in vitro* by switching the temperature to 37°C [12]. This dimorphism is essential for virulence in the mammalian host [13, 14] and is also found in other human pathogenic fungi such as *Blastomyces dermatitidis*, *B. gilchristii*, *Histoplasma capsulatum*, *Paracoccidioides brasiliensis*, *P. lutzii*, *Coccidioides immitis* and *C. posadasi*. However, all of these other dimorphic fungi are members of the order Onygenales, phylogenetically distant from *Sporothrix* in the Ophiostomatales [15]. The long genetic distance between these two orders suggests that thermo-dimorphism is a convergent phenotype shared by only a few members of these two orders. Genes such as histidine kinase (*drk1*) regulates the transition of mycelium to yeast and consequently the maintenance of virulence in *B. dermatitidis and H. capsulatum* [16]. This gene, also identified in *S. schenckii,* shows 65% of identity with its ortholog in *B.dermatitidis* and seems to be highly expressed during the yeast stage [17].

Besides dimorphism and thermo-tolerance, current knowledge about virulence factors of *Sporothrix* remains scant. The cell surface of pathogenic fungi plays a key role in the host-fungus interplay, mediating various processes associated with pathogenesis. The fungal cell wall is mainly composed of glycoconjugates: structural polysaccharides such as chitin and β-glucans, and cell wall glycoproteins [18]. Few proteins and glycoconjugates have been identified so far in the *S. schenckii* cell wall and their relevance for the host-fungus interaction and stimulation of the host immune system was reinforced by recent studies [19, 20]. However, the identity of the enzymes involved in biosynthetic pathways of cell wall components is still lacking. Another cell wall virulence factor, melanin, was found in *S. schenckii* conidia and yeast cells being produced *in vitro* or *in vivo* during infection [21]. Melanin pigments protect the fungus from the mammalian host’s innate immune responses providing resistance to oxidizing agents and fungal cell death during phagocytosis [22, 23].

Members of the pathogenic lineage in *Sporothrix* seem to behave in the host remarkably different from Ophiostoma species, suggesting a fundamental habitat shift from a plant- to a mammal-associated life style [24]. Remarkably, most fungi from the order Ophiostomatales live in association with bark beetles in woody plants, displaying adaptation strategies for insect transmission that are very different from those of *S. schenckii* and their relatives [2, 25]. The main biological questions of this work revolve around the dimorphic and pathogenic status of the two *Sporothrix* species, which are phenotypically similar to human/animal pathogenic Onygenales but philogenetically closely related to plant-associated Sordariomycetes. To address these questions, we performed a comparative genomic analysis of the pathogens with 14 other fungi, either dimorphic pathogens or plant-associated Sordariomycetes. Of these, we chose the closest relative to the *Sporothrix* lineage, *Grosmannia clavigera*, for more detailed comparison. *G. clavigera* is a tree-pathogenic and insect-associated fungus from a related genus from the Ophiostomatales order [26]. It is a haploid filamentous Ascomycete and a symbiont of the bark beetle *Dendroctonus ponderosae*, which affects commercial conifer forests, parks, protected areas and urban forests across North America [27]. These genomic analyses allowed us to identify the core genes for general and secondary metabolism as well genes related to autophagy, adhesion, cell wall assembly and melanin biosynthetic processes. We have also shown that genomic adaptation in the *Sporothrix* lineage has led to expansion of some protein domains and lack of genes associated with plant biomass decay when compared to other Sordariomycetes, which can be interpreted as an adaptation from plant to an animal associated life style.

## Results and discussion

### Genomes features, assemblies and synteny

**Figure 1:**
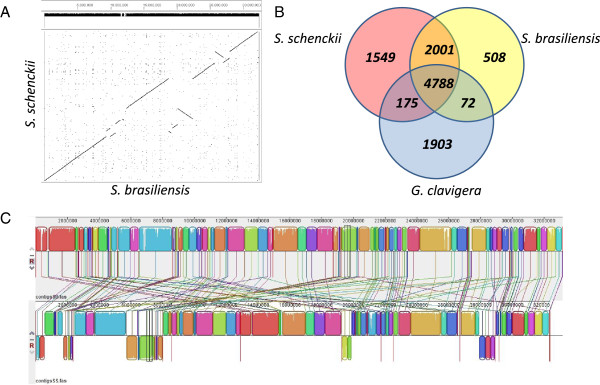
**Genomic alignments, synteny and homology of** ***S. schenckii*** **and** ***S. brasiliensis*** **. A)** Dot-plot of *S. schenckii* and *S. brasiliensis* using ordered scaffold sequences. **B)** Predicted proteins in *S. schenckii* and *S. brasiliensis* were compared with the predicted proteins of *G. clavigera*. The Venn diagram was built using minimum query/subject coverage of 50% and e-value of E ≤ 1×10^−20^. **C)** Genomic alignments of *S. schenckii* (bottom) and *S. brasiliensis* (top) showing chromosomal inversions in the genomes of these pathogens.

The *S. schenckii* and *S. brasiliensis* genomes were each pyrosequenced to ~20x coverage. The *S. schenckii* genome (strain 1099–18) yielded 16 scaffolds with N50 of 4.3 Mb, containing 237 contigs comprising a total size of 32.4 Mb. The *S. brasiliensis* genome (strain 5110) yielded 13 scaffolds with N50 of 3.8 Mb, containing 601 contigs, had a total genome size of 33.2 Mb, and shared similar genomic characteristics with *G. clavigera* [26] (Table 1). Telomeric repeats (TTAGGG/CCCTAA)_n_ were found at 5’ or 3’ terminal ends of 5 out of 13 scaffolds in the *S. schenckii* and 7 out of 13 scaffolds in the *S. brasiliensis* genome. Terminal repeats were found in both ends of 1 and 3 scaffolds of *S. schenckii* and *S. brasiliensis* respectively, revealing the presence of complete linear chromosomes. Pair-wise genome alignments showed that both *Sporothrix* species are highly syntenic sharing 97.5% average sequence identity (Figure 1A). According to the genomic alignments long inverted segments were found in the two *Sporothrix* genomes (Figure 1A-C). *S. schenckii* and *S. brasiliensis* were predicted to have 10,293 and 9,091 protein coding genes respectively, similar to other Eurotiomycetes and Sordariomycetes, and slightly higher than *G. clavigera* (Table 1). The G + C content in *S. schenckii* and *S. brasiliensis* genomes is one of the highest in Ascomycota. *S. schenckii* and *S. brasiliensis* genomes display 62% of G + C contents in both species, which is considerably higher than *G. clavigera* (53.4%) [26] and 50–52% in most other fungi in Pezizomycotina [28]. We detected similar distributions of transcript lengths, but we found less introns per gene in *Sporothrix* genomes than in those of *G. clavigera.* The tRNA contents revealed a great discrepancy among the analyzed fungi; *G. clavigera* harbors at least 2-fold more tRNAs than *Sporothrix* genomes (Table 1). We analyzed the homology relationships among fungi from the Ophiostomataceae family, comparing the gene content of *S. schenckii*, *S. brasiliensis* and *G. clavigera* by Bidirectional-best Blast Hits (BBH). A total of 4,788 genes were found in all three genomes and 2,001 were found to be *Sporothrix*-restricted genes, indicating a high content of specific genes in the *Sporothrix* lineage. A total of 1,549 and 508 genes were considered orphan sequences in *S. schenckii* and *S. brasiliensis*, respectively (Figure 1B). We have performed the comparative analysis of core genes involved in general and secondary metabolism, as well genes involved in transport and catabolism showing a high degree of conservation when compared to those present in other Ascomycetes (Additional file 1: Text 1). Genomes from *S. schenckii* and *S. brasiliensis* were deposited in the Genbank under respectively accession numbers: AXCR00000000 and AWTV00000000.

**Table 1:** ***Sporothrix*** **genome characteristics**

Characteristic	***S. schenckii***	***S. brasiliensis***	G. clavigera*
Genome size	32.4 Mb	33.2 Mb	29.8 Mb
Coverage	17X	20X	64X
Supercontig number	16	13	18
N50 supercontig	4.3 Mb	3.8 Mb	1.2 Mb
G + C content	62%	62%	53.4%
Protein coding genes	10,293	9,091	8,314
Median Transcript length	1,522 bp	1,602 bp	1,641 bp
Introns per gene	1.0	1.1	1.9
Median Intron length	91.2 bp	123.4 bp	70 bp
Median Intergenic distance	1,530 bp	1,913 bp	1,466 bp
tRNA	139	140	268

*Genomic information collected according previously published *G. clavirera* genome [26].

### Phylogenomic analysis

**Figure 2:**
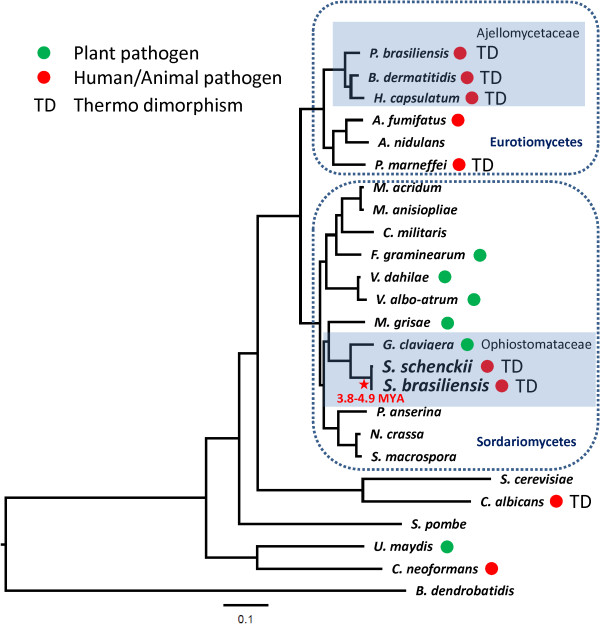
**Phylogenomic relationships of** ***Sporothrix*** **species and other thermodimorphic fungal pathogens.** The phylogenetic tree was constructed from an alignment of 153,436 amino acids of 395 orthologous protein clusters. The tree was inferred by Maximum Likelihood method implemented in RAxML and Dayhoff amino acid substitution was used as the best protein substitution model. The tree was calibrated with the origin of the Ascomycota clade around 500–650 MYA.

A total of 395 orthologous protein clusters were identified by BBH after searching 25 fungal genomes, including Ascomycetes, Basidiomycetes and Chytridiomycetes (Additional file 2: Table S1). A Maximum Likelihood phylogenomic tree was generated using a 153,436 amino acids position alignment and calibrated with the origin of Ascomycota clade around 500–650 MYA. The phylogenomic tree, as expected, placed *S. schenckii* and *S. brasiliensis* in a monophyletic clade closest to *G. clavigera* being apart from other Sordariomycetes (Figure 2). According to the phylogenomic tree, *S. schenckii* and *S. brasiliensis* diverged about 3.8-4.9 MYA suggesting a recent event of speciation in the genus *Sporothrix*. Additionally, evolutionary origin of the ophiostomatoid fungi was dated to 69.1-89.9 MYA, being highly divergent from the plant pathogen *G. clavigera* (Figure 2). The divergence time varied across sister species of fungal pathogens along the Ascomycota phylum, such as *C. immitis* vs. *C. posadasii* diverged about 5.1 Mya [29] and *P. brasiliensis* vs. *P. lutzii* about 11 to 32 Mya [30].

### Mitochondrial genomic comparisons

**Figure 3:**
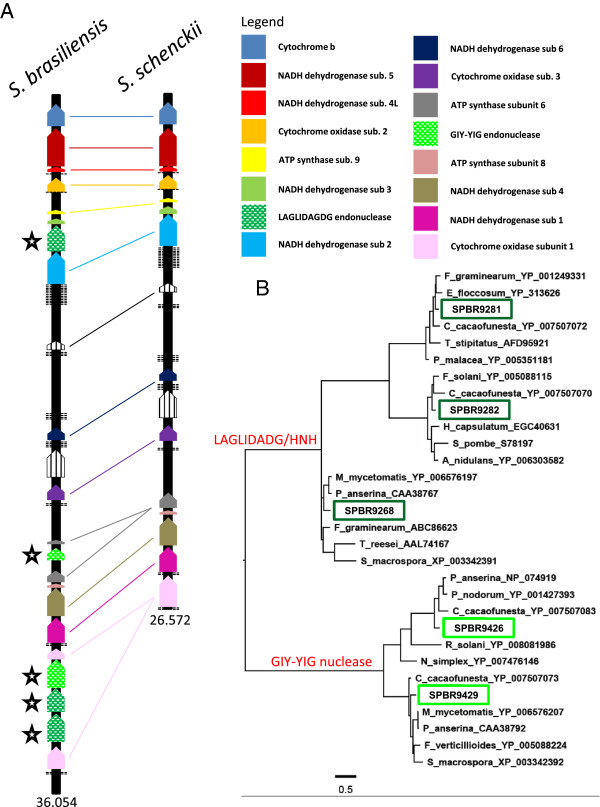
**Comparative analysis of mitochondrial genomes of** ***S. schenckii*** **and** ***S. brasiliensis*** **. (A)** Gene content and order in mitochondrial genomes of *S. schenckii* vs. *S. brasiliensis* showing high synteny despite the considerable difference in size. Insertions of LAGLIDADG and GIY-YIG intron I type homing endonucleases in *S. brasiliensis* are present inside cytochrome C oxidase 1, ATP synthase subunit 6 and NADH dehydrogenase subunits 2 and 3 genes. **(B)** Phylogenetic analysis was performed using the five LAGLIDADG or GIY-YIG elements found in *S. brasiliensis* showing no pattern of ancestry with other close related species.

The mitochondrial genome assembly of *S. schenckii* strain 1099–18 is 26.5 Kb in size and shares 99-100% average sequence identity and 97-100% coverage in comparison to that of previously published *S. schenckii* strains ATCC 10268 (AB568599) and KMU2052 (AB568600) (data not shown). The mitochondrial genome assembly of *S. brasiliensis* strain 5110 spans 36 Kb but covers only 71-75% of the three *S. schenckii* mentioned genomes before. Despite the high similarity between the two analyzed mitochondrial genomes (99% of identity), *S. brasiliensis* harbors parasitic group-I intron encoding homing endonucleases (HE’s) which is responsible for the higher mitochondrial genome size in this species. Those elements were detected in the cytochrome C oxidase 1, ATP synthase subunit 6 and between NADH dehydrogenase subunits 2 and 3 ORF’s (Figure 3). These HE’s found in the *S. brasiliensis* mitochondrial genome were classified into two families according Interpro domain screening: LAGLIDADG and GIY-YIG. Mitochondrial LAGLIDADG HE’s from *S. brasiliensis* (SPBR09268, SPBR09281 and SPBR09282) shared 75%, 84% and 78% of identity to *Madurella mycetomatis* (YP_006576197), *Fusarium graminearum* (YP_001249331) and *F. solani* (YP_005088115), respectively. The *S. brasiliensis* mitochondrial GIY-YIG HE (SPBR09426 and SPBR09429) is highly conserved among other Sordarimycetes, sharing 72% and 77% of identity to *Podospora anserina* (NP_074919) and *Ceratocystis cacofunesta* (YP_007507073), respectively . *C. cacofunesta* contains 37 intronic ORFs, thus being responsible for one of the largest mitochondrial genomes among Sordariomycetes [31]. Fungal mitochondrial genomes present a constant genetic mobility, probably due to the activity of group-I intron encoding homing endonucleases. Mitochondrial introns and their ORFs have been associated with mitochondrial parasitism and genomic size changes thus causing genomic instability, which was reported before in *S. cereviseae*, *P. anserina*, *Neurospora crassa*, *Ophiostoma* and *Aspergillus* [32–36]. According to the phylogenetic tree, no common ancestor was found in Sordariomycetes class, suggesting an independent or convergent evolution of group-I intron encoding LAGLIDADG and GIY-YIG elements in Ascomycota (Figure 3).

### Transposable elements expansions in *S. brasiliensis*genome

Transposable elements (TEs) comprise 0.34% and 0.62% of the *S. schenckii* and *S. brasiliensis* genomes, respectively (Table 2). Fungal genomes contain substantially different amounts of repetitive DNA sequences. The assembled genome of *Magnaporthe oryzae* contains 10.8% of repetitive DNA sequences, in *M. grisea* it is 4.2%, in *N. crassa* is 10% and in *S. cerevisiae* almost 6% [37–40]. Differences in the TE contents are observed even between closely related species, e.g. in the genus *Paracoccidioides*. In *P. brasiliensis* TEs correspond to 8-9% of the genome and twice this amount in *P. lutzii* (16%) [41]. Although less common, lower TE contents have been described in other fungi, for example 0.48% of *Trichoderma* and 0.1% of *Fusarium graminearum* genomes assemblies [39, 42].

**Table 2:** **Transposable element composition in** ***Sporothrix*** **genomes**

Type	***S. schenckii***	***S. brasiliensis***
LTR - Copia-like	3	3
LTR - Gypsy-like	12	57
LTR - BelPao-like	1	0
LINE	14 (1)^a^	7
Tc1/mariner-like	9	6
hAT-like	12	9
Mutator-like	1	4
PiggyBac –like	1	0
Helitron	0	4
Total elements	53	90
Percent of assembly	0.34%	0.62%

^a^The number of potentially functional elements is shown in parentheses.

**Figure 4:**
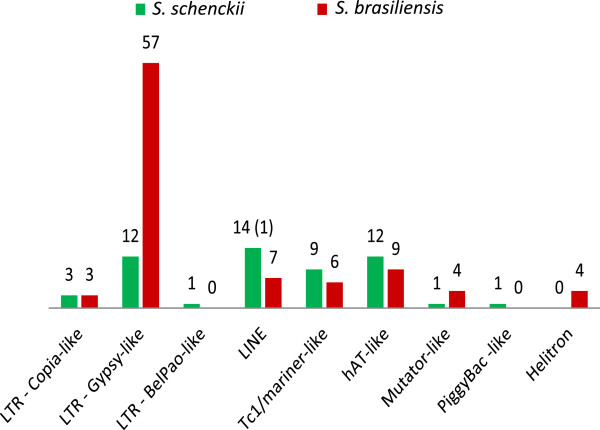
**Transposable element content in** ***Sporothrix schenckii*** **and** ***S. brasiliensis*** **genomes.** The number of potentially functional elements is shown in parentheses.

Despite their lower content in *Sporothrix* genome, all classes of TEs have been detected with large variation in number and diversity between *S. schenckii* and *S. brasiliensis*. Two major types of retrotransposons, *LINEs* and LTRs, were found in *S. schenckii* and *S. brasiliensis*, but no *SINE* elements were found (Table 2). In *S. brasiliensis,* a five-fold expansion of *Gypsy*-like elements was observed compared to *S. schenckii* and both genomes contain more *Gypsy*-like elements than *Copia*-like elements. We have observed a 4 and 19 fold-change between *Gypsy*-like and *Copia*-like elements for *S. schenckii* and *S. brasiliensis* respectively, as usual for fungal genomes [38]. The overall 2-fold expansion of repetitive sequence content between *Sporothrix* species may reflect an expansion of retrotransposons in the *S. brasiliensis* genome or either a contraction in that of *S. schenckii* (Figure 4). According to population genomics studies and mating type distribution, the *S. brasiliensis* population appears to be clonal and no recombination events were detected [43]. We observed a predominance of a single mating clone in epidemics of sporotrichosis and no or limiting sex could favor the accumulation of repetitive elements in microorganisms genomes [44, 45]. Assexual propagation could lead extinction due inability to control proliferation of vertically transmitted TEs, as accumulation occurs due to the inefficiency of purifying selection in clonal species [46].

Equivalent numbers of DNA transposons were found in *S. schenckii* and *S. brasiliensis* genomes. *hAT-like* elements were the most prevalent in both species (Table 2). Transposons from *Tc1/mariner* and *Mutator* superfamilies were also found in both genomes. Four copies of *Helitrons* were identified in *S. brasiliensis* while a *PiggyBac*-like element was detected only in *S. schenckii*. Almost all TEs identified in *Sporothrix* genomes corresponded to defective and truncated copies, with exception of one *LINE*-like element found in *S. schenckii*, suggesting that *Sporothrix* species are able to control their proliferation. This finding suggests that TEs are present in *Sporothrix* but with low transposition activity. We discuss why this could be below.

### Protein family expansion and contraction in the *Sporothrix*lineages

**Figure 5:**
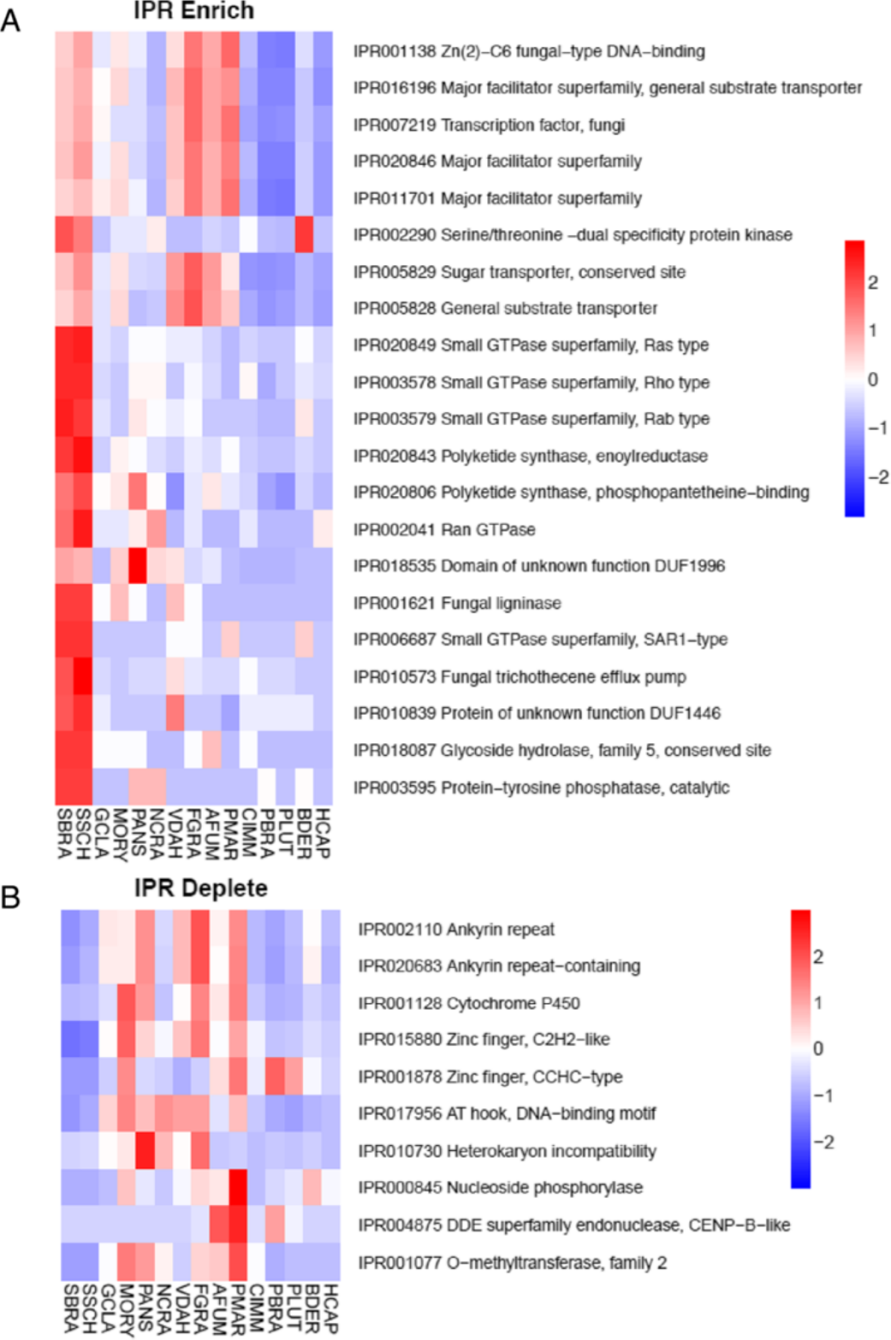
**Interpro domains most enriched (A) or depleted (B) in** ***Sporothrix*** **lineages compared to other thermo dimorphic fungi and close related Sordariomycetes.** SBRA - *S. brasiliensis*, SSCH – *S. schenckii*, GCLA – *G. clavigera*, MORY – *M. oryzae*, PANS – *P. anserina*, NCRA – *N. crassa*, VDAH - *V. dahliae*, FGRA – *F. graminearum*, AFUM – *A. fumigatus*, PMAR – *P. marneffei*, CIM – *C. immitis*, PBRA – *P. brasiliensis*, PLUT – *P. lutzii*, BDER – *B. dermatitidis*, HCAP – *H. capsulatum*. The reported p-values were used for multiple testing using q-value.

Gene duplications are an important source of evolutionary innovation and new gene copies can evolve new adaptive functions shaping an organism’s gene content. The differences among gene families have been related to emerging processes due to differential degrees of genetic drift, and thus the effectiveness of selection, acting on genomes [47]. The pathogenic phenotypes of *S. schenckii* and *S. brasiliensis* could result of expansion in specific gene families that confer advantages in the interaction with human/animal hosts. On the other hand, gene families that ate necessary for the plant-associated lifestyles of other Sordariomycetes could be contracted. To test these hypotheses, we have compared the genomes from closely related Sordariomycetes and dimorphic fungal pathogens. Changes in *S. schenckii* and *S. brasiliensis* gene families were inferred based on domain expansions or contractions assigned by Interpro, Pfam and SMART databases and statistically tested by hypergeometric comparisons (P < 0.05 - Figure 5, Additional file 3: Figure S2, Additional file 4: Figure S3 and Additional file 5: Figure S4) and the reported p-values were used for multiple testing using q-value.

**Figure 6:**
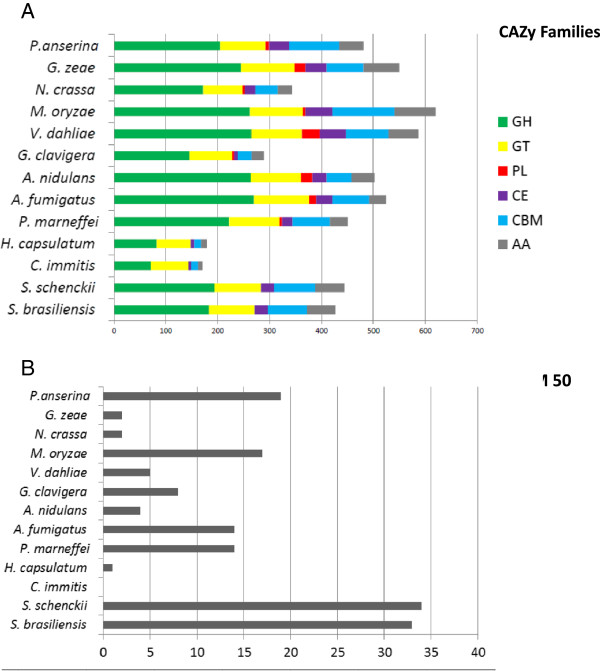
**Gene expansion/contraction of carbohydrate-active enzymes (CAZy) in the genus Sporothrix. (A)** Overall comparison of Glycoside Hydrolases (GHs), Glycosyl Transferases (GTs), Polysaccharide Lyases (PLs), Carbohydrate Esterases (CEs), Auxiliary Activities (AAs) and Carbohydrate-Binding Modules (CBMs) against other thermo dimorphic fungi and closely related Sordariomycetes. **(B)** CBM 50 gene family is highly expanded in the *Sporothrix* lineage.

We have not observed the enrichment of peptidases genes in *Sporothrix* lineage, specifically the MEROPS families M35 or M36, which are expanded in dimorphic fungal pathogens as adaptation to mammalian hosts [29, 41]. On the other hand, we observe a lack of polysaccharide lyase genes which are associated with decay of plants (CAZy PL family) when compared to other Sordariomycetes (Figure 6A, Additional file 2: Table S5). The subfamilies PL1, PL3 and PL4 are broadly distributed among Sordariomycetes, but were not observed in the *Sporothrix* species. Interestingly loss of plant degrading enzymes were also observed in other dimorphic fungal pathogens such as *H. capsulatum* and *C. immitis* (Figure 6A, Additional file 2: Table S5), which has also been previously interpreted as adaptation from plants to animals [29, 41]. As an alternative for the absence of PL proteins, it may be hypothesized that *Sporothrix* may digest pectin by using polygalactouronase (CAZy GH28) as a replacement strategy. Additionally, we didn’t detect any GH72 genes in *Sporothrix* genomes although those genes were found in all remaining analyzed fungi (Additional file 2: Table S5).

**Figure 7:**
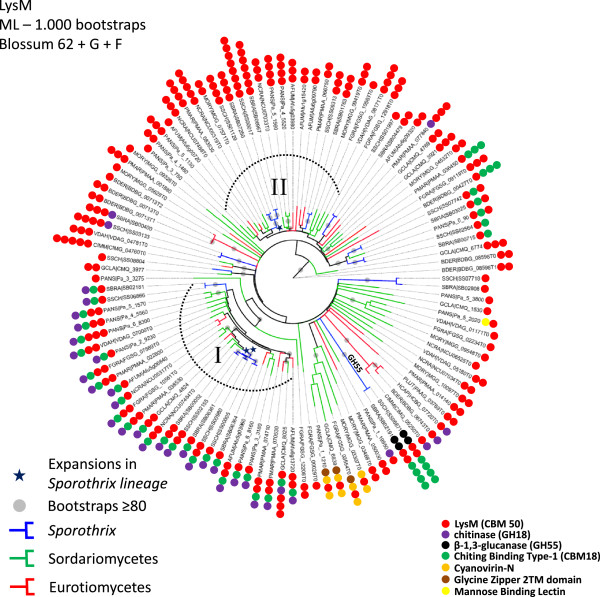
**Unrooted maximum likelihood tree revealing LysM expansions in the** ***Sporothrix*** **lineage.** Two major expansions were detected: The first duplication is related to genes containing single or multiple repetitions of LysM domains (CBM50), chitin binding module type 1 (CBM18) plus glycoside hydrolase (GH18) – clade I. The second event of duplication was observed in genes presenting multiple copies of LysM (CBM50), and is evidence of recent intergene duplication of this domain within paralogous - clade II.

LysM domain-containing proteins display carbohydrate binding modules, usually 42–48 amino acids residues in length, found in prokaryotes and eukaryotes. These proteins are classified in the CAZy database as CBM family 50 and harbor N-acetylglucosamine (GlcNAc) binding characteristics. According to PFAM and CAZy counts, a marked expansion of the LysM domain PF01476 (CAZy CBM family 50) was detected in the *Sporothrix* lineage. The phylogenetic tree clearly showed highly expanded branches in the *Sporothrix* lineage when compared to other Sordariomycetes and thermo dimorphic fungal pathogens (Figure 7). Moreover, the comparative analysis of CAZy CBM family 50 also revealed a high expansion of this domain (Figure 6B, Additional file 2: Table S5). Recent events of duplications of LysM homologs subsequent to a speciation event were detected in the *Sporothrix* lineage using reconciled tree approach (Additional file 6: Figure S1). The first duplication (Figure 7, clade I) is related to genes containing single or multiple repetitions of LysM domains, chitin-binding module type 1 (CBM18) plus glycoside hydrolase (GH18). The second event of duplication was observed in genes harboring multiple copies of LysM, providing evidence of recent intergene duplication of this domain within paralogues (Figure 7, clade II). The structure of *Sporothrix* LysM domain-containing genes are presented separately in Additional file 3: Figure S2.

Chitin is a linear polymer of β-(1,4)-linked GlcNAc, and is one of the major components of fungal cell wall. The absence of chitin in mammalian cells makes this polymer a potential targets for the innate immune system [48]. Chitin can be recognized by mammalian cells, and is bound and degraded by chitin-binding proteins GH18, playing an important role in inflammation and innate and adaptive immunity based on their modulation on various disease states [49]. Chitin can mask immune recognition by blocking dectin-1-mediated interaction with fungal cell walls [50]. Also, chitin modulates epithelial immunity of the skin expressing high levels of cytokine and chemokine and increases TLR4 expression on keratinocytes [51]. Possibly these proteins are present to bind the own *Sporothrix* chitin exposed upon cell damage, and in this way it may protect itself against recognition of this polymer by keratinocytes. LysM effector (Ecp6) from *Cladosporium fulvum* was characterized as a virulence factor of this phytopathogenic fungus on tomato plants. Carbohydrate binding assays have shown that Ecp6 specifically binds to chitin, the major constituent of the fungal cell wall, acting as a PAMP upon recognition by the plant during fungal invasion. The presence of chitin-binding effector Ecp6 in the apoplast masks the perception of chitin by plant receptors preventing the activation of defense responses [52]. In addition significant expansion of the LysM domain was detected in dermatophytes, compared to the thermodimorphic fungi [53]. Dermatophytosis and sporotrichosis are characterized as cutaneous mycoses and the infection is acquired after contact and/or trauma of skin. LysM domain containing proteins have not been characterized as virulence factors in human or animal fungal pathogens so far, and the role of those proteins requires further investigation and could be a novel mechanism for fungal evasion in mammalian host tissue.

Small GTPases are an independent superfamily of GTP-binding proteins, sharing a common enzymatic activity and producing GDP by the hydrolysis of GTP, and play pivotal roles in cell division and signaling, vesicle fusion and protein synthesis [54]. These proteins are also involved in filamentation, mating, growth at 37°C and virulence in *C. neoformans* [55, 56]. Significant expansions in Ras, Rho and Rab Small GTPase superfamilies (IPR020849, IPR003578 and IPR003579) were observed in the *Sporothrix* lineage when compared to other Ascomycetes (Figure 5, Additional file 2: Table S4). Judging from the phylogenetic trees of small GTPases, the majority of branches harboring *Sporothrix* homologues suggests a higher diversity among the Ascomycetes we analyzed (Additional file 4: Figures S3, Additional file 5: Figure S4, Additional file 7: Figure S5). Reconciliation of small GTPases gene trees and species tree do not indicate *Sporothrix*-specific duplications, that instead independent gene losses in other species explain the increased copy number in *Sporothrix* (Additional file 8: Figure S6, Additional file 9: Figure S7, Additional file 10: Figure S8). An alternative hypothesis is that the *Sporothrix* Ras, Rho and Rab genes have duplicated recently and rapidly diverged creating the observed long branch lengths. In addition we detected highly supported clades containing *Sporothrix* and other pathogenic dimorphic fungi, suggesting convergent evolution of small GTPases, reinforcing the high plasticity of signal transduction in the *Sporothrix* lineage (Additional file 4: Figure S3, Additional file 5: Figure S4, Additional file 7: Figure S5). To date, such a high diversity of Small GTPase proteins as found in the *Sporothrix* lineage has not been reported from any other class of fungi.

**Figure 8:**
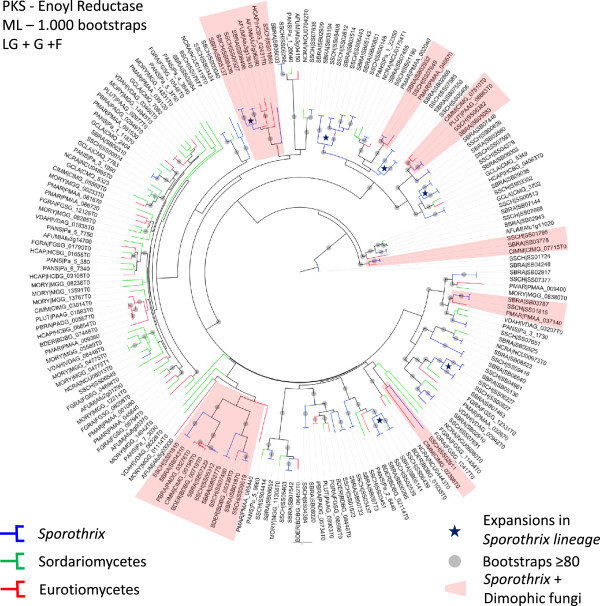
**Unrooted maximum likelihood tree of Polyketide synthase (PKS) enoylreductase (IPR020843) family showing expansions in the** ***Sporothrix*** **lineage.** Paralogous duplications are displayed in the related branches suggesting vast genomic apparatus of PKS containing genes in the *Sporothrix* lineage. Clades harboring *Sporothrix* and dimorphic fungi are displayed in red.

Another group that was expanded by INTERPRO and SMART domain counts in the *Sporothrix* lineage is the polyketide synthase (PKS), enoylreductase (IPR020843) family (Figure 5, Additional file 2: Tables S3, S4). Polyketides comprises diverse fungal secondary metabolites such as antibiotics, pigments, and mycotoxins that are formed from simple carbon precursor acids catalyzed by polyketide synthases (PKSs) [57]. Filamentous fungi are producers of polyketide metabolites, several of which of pharmacological or agricultural interest [58, 59]. Fungal PKSs are in general a linear succession of ketosynthetase (KS), acyltransferase (AT), dehydratase (DH), enoyl reductase (ER), ketoreductase (KR), acyl carrier protein (ACP), and thioesterase (TE) domains [60]. ER domains reduce enoyl groups to alkyl groups (saturated) during production of secondary metabolites. Among fungal genomes, few potential PKS orthologous genes are shared, even between closely related taxa [61]. We identified various paralogous duplications in the phylogenetic analysis of PKS-containing protein, and the *Sporothrix* lineage appears to have of PKS-encoding genes that is at least 3-fold larger than that of the other species analyzed (Additional file 2: Table S4, Figure 8). This was confirmed using the reconciled approach of PKS gene tree with the species tree analyzed (Additional file 10: Figure S8). Convergent branches linking *Sporothrix* and other pathogenic dimorphic fungi were also observed. We identified discontinuous distributions of PKS homologs among the analyzed fungal species, with low bootstrap values, that can be explained by gene duplication, divergence, and gene loss [61]. We also identified expanded clades harboring *Sporothrix* and dimorphic fungi with high branch support values, suggesting a great diversity of this protein family.

Another large expansion found in the *Sporothrix* lineage is a fungal trichothecene efflux pump (Pfam PF06609), an evidence of detoxification via mycotoxin pump [62]. Apart from that, fungal genomes generally harbor a lower content of Leucine-rich repeat (LRR) proteins than other ophistokonts [63]. Gene expansions of several LRR superfamilies were identified in the *Sporothrix* lineage (Additional file 2: Tables S3, S4). LRR proteins expansions are commonly found in eukaryotic parasites such as *Trypanosoma* and *Giardia* [64, 65] *.* These domains consist of 2–45 motifs of 20–30 amino acids in length providing a structural framework for protein-protein interactions. LRR proteins are involved in a variety of biological processes and are source of genetic variation for the ongoing process of antigenic variability in pathogens [66, 67]. In addition expansion of LRR proteins was described for *Candida* species as a virulence factor [68]. The role of these proteins should be further investigated in the genus *Sporothrix*.

### Cellular processes and dimorphism

The basic mechanisms of DNA repair in *Sporothrix* do not deviate from the eukaryotic consensus, as expected, but neither *S. brasiliensis* nor *S. schenckii* seem to have a homolog to the *Neurospora crassa* RIP-defective (RID) methyl-transferase [69]. This suggests that RIP-like mutation patterns found in transposable elements in this fungus are not generated by the classical, RID-dependent mechanism. This is not a surprise, given that at least one previous report [70] pointed to the possibility of alternative pathways for repetitive DNA quelling via RIP. This is in contrast to other Sordariomycetes and even more distantly related members of the Ascomycota, such as *P. brasiliensis* and *H. capsulatum*, for which the tBLASTn tool yielded hits with high homology. Since the number of degenerate TEs and overall evidence of transposon activity in *Sporothrix spp.* genomes is low, it would be interesting to identify which pathway is responsible for TE suppression in this genus instead of RIP. We also note the absence of a *mus-18* homologue for the alternative, UV damage-related nucleotide excision repair pathway [71], found in *Neurospora crassa*.

Among transcription factors, the recent discovery of the Ryp1 protein in *H. capsulatum* [72] is of interest for the study of adaptive processes in fungi. Ryp1 and its homologues in *C. albicans* and *S. cerevisiae* [73] are all implicated in morphogenetic changes of their respective species in response to environmental stimuli. We have also found an ortholog of Ryp1 in *Sporothrix*. Given that Ryp1 and the hybrid histidine-kinase Drk1 are two determinants of mold-to-yeast transition in dimorphic fungi, and that *Sporothrix* also has homologues for the latter [16], it seems reasonable to speculate that both also have been coopted by this genus to coordinate dimorphic transition.

### Cell wall assembly

The cell wall of fungi is a dynamic organelle, which is constantly adjusted depending on environmental insults. Fungal cell wall components are considered relevant for virulence, have antigenic properties and participate in the modulation of the host immune response by being recognized by innate immunity receptors [14]. Current models propose an arrangement of several stratified layers composed of structural polysaccharides, mainly β-glucans and chitin, proteins and glycoproteins, generally known as mannoproteins, and other minor components. Cell wall proteins (CWP) are either covalently linked to the cell wall β (1,6)-glucans by a glycophosphatidylinositol (GPI) moiety or linked via an alkali sensitive linkage to β (1,3)-glucans. The sugar moieties in CWP are *N*– and/or *O*- linked to the protein core [74]. Despite the importance of those components, little is known about the surface protein composition of *S. schenckii* and *S. brasiliensis*.

#### a) Adhesins and/or cell surface proteins

Previous studies searching for adhesins and antigens in cell extracts of *Sporothrix* demonstrated the presence of a main antigen on both species, known as Gp70, a secreted antigen that is also present on the cell surface acting as an adhesin [75–77]. The genomes of *S. schenckii* and *S. brasiliensis* were investigated with different predictors to ascertain the presence of these cell wall components.

**Figure 9:**
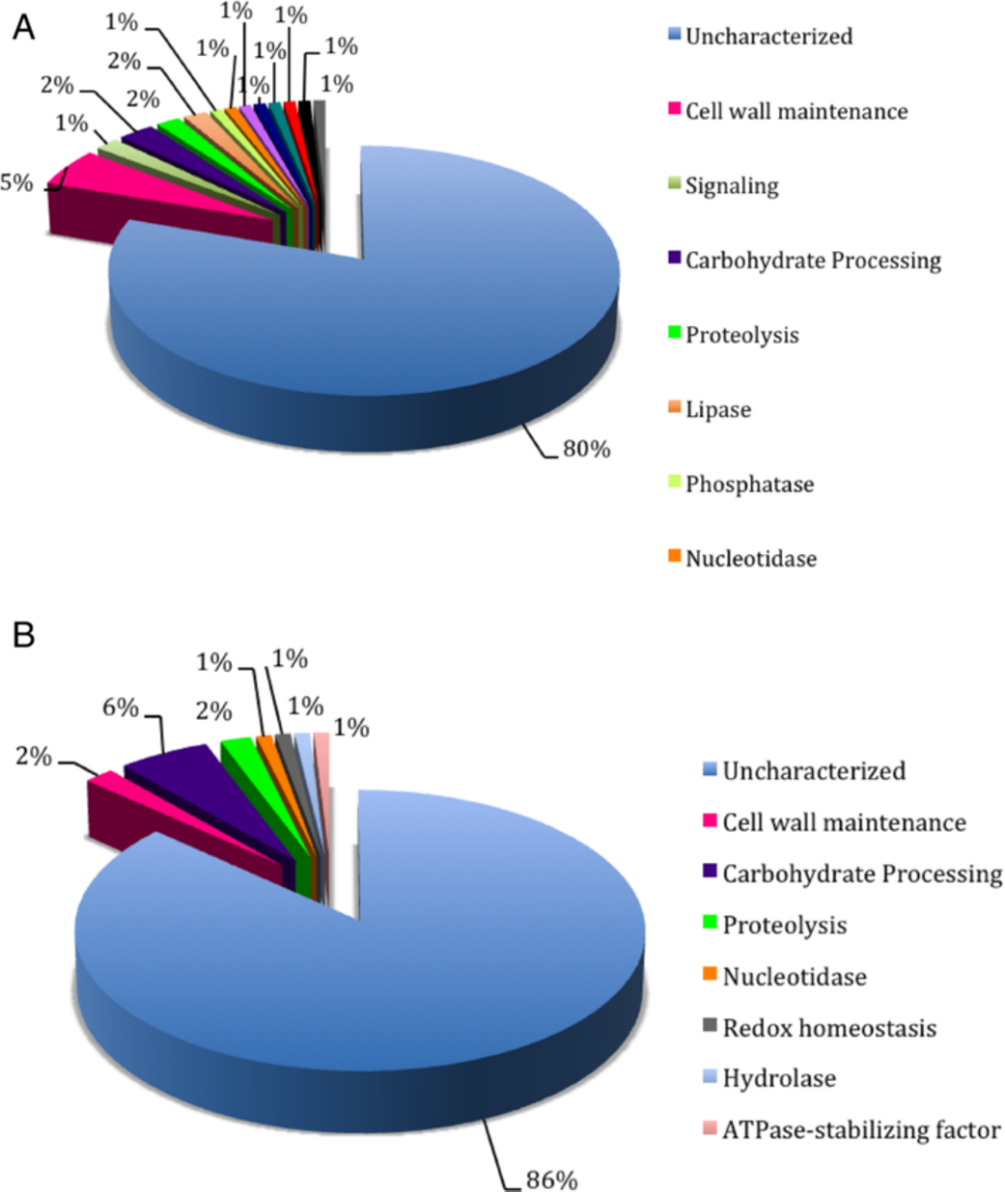
**Putative adhesins identified and/or cell wall GPI-anchored proteins in** ***S. schenckii*** **(A) and** ***S. brasiliensis*** **(B) predicted by ProFASTA and FungalRV.** Putative adhesins were classified by their biological function using Gene Ontology (GO) and InterPro databases.

An *in silico* comparative analysis was performed in order to determine the putative adhesins and/or cell surface proteins bearing a GPI-anchor. According to ProFASTA and FungalRV, *S. schenckii* harbors 68 and 61 surface proteins, respectively, that have adhesin properties (n = 129). Of these, 12 were found by both predictors (Additional file 11: Figure S9A, Additional file 2: Table S6) totalizing 117 unique predicted proteins in the cell wall of *S. schenckii*. For *S. brasiliensis* we have identified 54 and 63 cell wall proteins (n = 117), respectively, 11 of which were predicted by both algorithms. For this species, a total of 106 unique predicted proteins (Additional file 11: Figure S9B, Additional file 2: Table S7). The protein sequence relative to the previously proven cell surface/adhesin Gp70 was not predicted to possess this function or to be present in the surface location by any of the predictors used [77]. Previous studies described several other important, non-classical surface proteins present in other fungi but which were not recognized in *Sporothrix* by FungalRV and ProFASTA [78–80]. The major classes of proteins predicted in *S. schenckii* and *S. brasiliensis,* by both ProFASTA and FungalRV, are currently annotated either as hypothetical proteins or belonging to a protein family with unknown function (Figure 9). This indicates that proteomic studies are needed to validate the expression of such proteins and biochemical functional studies are necessary to clarify their role on the fungal cell surface. The cell wall proteins and/or adhesins predicted for these species were blasted against 11 fungi for comparative analysis, and each protein was blasted against the two *Sporothrix* species (Additional file 10: Table S8). Eight proteins were found exclusively in *S. brasiliensis* and eight proteins were present only in *S. schenckii.* Sixteen *Sporothrix*-specific proteins were annotated as hypothetical proteins. None of the proteins described are specific to the group of human pathogenic fungi, but interestingly there are some proteins putatively present in the cell wall of *Sporothrix* that share homology with those of plant and insect-associated fungi.

#### b) Glucan and chitin metabolism

In *S. schenckii*, β-glucans are major components of the cell wall [81, 82], and are present as alkali-insoluble and alkali-soluble glucans, containing predominantly β-(1,3)-linkages in both cases [82]. In *S. schenckii*, no genes related to the synthesis or degradation of β-glucans have been reported to date. Genomic data analysis revealed a single *FKS* ortholog in *S. schenckii* and *S. brasiliensis* genomes, as well as single orthologs in the genomes of the other 14 fungal species studied here (Additional file 11: Table S9). No genes related to the synthesis of either β-1,6- or β-1,4-glucans were identified, although hydrolase orthologs for the three types of β-glucan linkages in the *S. schenckii* cell wall, are present in both genomes (Additional file 11: Table S9).

Chitin synthesis in fungi is a rather complex process, regulated by multigene families encoding chitin synthase isoenzymes, whose activities may be spatially regulated to fulfill the multitude of roles ascribed to them [83]. Based on differences in regions of high sequence conservation, chitin synthases have been attributed to seven classes [83, 84], whose functional implications are not yet clear in all cases. Despite the low chitin content reported for *S. schenckii* [81], genomic analysis showed the presence of seven *CHS* genes, in *S. schenckii* as well as in *S. brasiliensis* genomes. A cluster analysis of their putative products, including 36 fungal chitin synthases, revealed that each of the translation products of the seven *CHS Sporothrix* genes identified in genomic databases (*CHS1* to *CHS7*), could be ascribed to each of the seven chitin synthase classes known (I to VII) (Additional file 12: Figure S10, Additional file 2: Table S10). It is worth noting that class III chitin synthases were thought to occurr exclusively in filamentous fungi [85]. In our analysis, genes for class III chitin synthases were found in the *Sporothrix* genomes as well as the genomes of other dimorphic fungi (Additional file 2: Table S10), an indication that class III fungal chitin synthases might be more widespread in fungi. Another interesting finding was the head-to-head arrangement in the *S. schenckii* and *S. brasiliensis* genomes of the *CHS4* and *CHS5* genes, whose putative translated products are class V and VII chitin synthases (Additional file 8: Figure S6, Additional file 2: Table S10). A similar arrangement was reported for genes coding for classes V and VII of chitin synthases in *P. brasiliensis*, *A. nidulans*, *C. posadasii* and *F. oxysporum* [84, 86–88]. The meaning of such arrangement is unclear, although a common transcriptional regulation for these genes has been suggested for *A. nidulans* and *P. brasiliensis* [84, 88, 89]. In agreement with the large number of *CHS* genes in the *Sporothrix* genomes, genomic analysis showed the presence of ten and nine chitinase genes, respectively, in the *S. schenckii* and *S. brasiliensis* genomes (Additional file 2: Table S10).

Polysaccharide synthesis and hydrolysis-related genes identified in *S. schenckii* and *S. brasiliensis* genomes correlate with the biochemical composition of the cell wall as reported by Previato *et al.* [81, 82]. It remains to be determined which individual synthase and/or hydrolase gene might be involved in shaping the yeast, mycelial and spore (conidial) walls of *Sporothrix* species, or even whether any of them might have any role in survival and would provide potential targets for the development of specific antifungal drugs.

#### c) Protein glycosylation

Glycoproteins are key components of the *S. schenckii* cell wall, but thus far little is known about their biosynthetic pathways [23, 24]. The genomes of *S. schenckii* and *S. brasiliensis* contained the orthologs involved in elaboration of the *N*-linked glycan core, its transference to proteins and in early trimming. These genes are also known to be involved in glycoprotein endoplasmic reticulum-associated degradation, a quality control system for proteins synthesized within the secretory pathway [90–92] (Additional file 2: Table S11). Furthermore, the genomes contain the putative orthologs encoding Golgi-resident glycosidases and glycosyltransferases that further modify *N*-linked glycans, generating both hybrid and complex *N*-linked glycans. The presence of a gene with significant similarity to those encoding the N-acetylglucosaminidase III (Additional file 2: Table S11), which adds the bisecting GlcNAc residue found in both hybrid and complex *N*-linked glycans [93], suggests an ability to elaborate more complex oligosaccharides than those found in *S. cerevisiae* [94]. Moreover, our analysis revealed genes encoding putative Golgi UDP-galactose and CMP-sialic acid transporters, suggesting the ability of these fungi to add these sugars to their glycans. *S. schenckii* and *S. brasiliensis* also contain an ortholog of *A. nidulans ugmA*, whose product generates the galactomannan-building sugar donor [95], and some putative galactosyltransferases (Additional file 2: Table S12). However, it remains to be addressed whether these enzymes participate in elaboration of glycoproteins and/or glycolipids. Sialic acid has previously been reported as a component of *S. schenckii* cell wall glycolipids [96], so it is likely that the putative Golgi CMP-sialic acid transporter is involved in modification of such lipids.

The biosynthetic pathway for *O*-linked glycans can also be predicted from the analysis of *S. schenckii* and *S. brasiliensis* genomes (Additional file 2: Table S13). Optimal characterization of *O*-linked glycans is via isolation from peptide-rhamnomannans [97]. They contain an α1,2.mannobiose core, an α1,2-glucuronic acid unit, and one or two rhamnose residues. The *S. schenckii* and *S. brasiliensis* genomes contain three putative glucuronosyl transferases that might participate in the elaboration of this *O*-linked glycan (Additional file 2: Table S13). Our genomic analysis could not find any obvious ortholog for rhamnosyl transferases, but *Sporothrix* contains all the required genes for synthesis of UDP-L-rhamnose (Additional file 2: Table S13) [98, 99], the sugar donor in the enzyme reaction catalyzed by rhamnosyltransferases [100]. Synthesis of GPI is quite conserved in eukaryotic cells and the *Sporothrix* genome contains all genes to elaborate this glycolipid (Additional file 2: Table S14).

### Melanin metabolism

Melanins are dark pigments formed by phenolic and indolic oxidation. These biopolymers are produced by a wide range of organisms, possibly contributing to the maintenance of several species throughout evolution [101]. In fungi, the expression of these pigments has been associated with virulence [102]. Fungi may synthesize melanin by several pathways: in pathogenic fungi, most commonly from endogenous substrate via the 1,8-dihydroxynaphthalene (DHN) pathway or the L-3,4-dihydroxyphenylalanine (L-DOPA) pathways [102]. The latter type is prevalent in Basidiomycetes. However, evidence of both pathways has been found in *S. schenckii* by means of specific substrate supplementation or drug-related inhibition of the respective pathways [22, 23, 103, 104].

Melanins are found in *S. schenckii* spores and yeast cells and are produced *in vitro* and during infection using hamsters as host model. Its detection has also been confirmed by immunofluorescence with monoclonal antibodies raised against *S. schenckii* melanin [21, 22]. In *S. schenckii*, melanin pigments can protect the fungus from the mammalian host’s innate immune responses providing resistance to killing by phagocytosis and oxidizing agents [22, 105]. Recently it was reported that *S. schenckii* and *S. brasiliensis* also produce pyomelanin, a melanoid pigment derived from the degradation of L-tyrosine via a 4-hydroxyphenylpyruvate dioxygenase [103].

Genomic comparison showed that both *S. schenckii* and *S. brasiliensis* possess enzymes with central roles in melanin synthesis via DHN and DOPA pathways, and also in pyomelanin synthesis. *Sporothrix schenckii* and *S. brasiliensis* loci which are postulated to be involved in the melanin biosynthesis pathway are described in Additional file 2: Table S15. We found 19 loci related to melanin biosynthesis in *S. schenckii* and 17 in *S. brasiliensis*. Homology with previously described melanin-related enzymes found in the sequence analyses are: pigment biosynthesis protein yellowish-green 1, polyketide synthase I and III, tetrahydroxynaphthalene/trihydroxinaphtalene reductase, scytalone dehydratase, laccase, tyrosinase and 4-hydroxyphenylpyruvate dioxygenase, as illustrated in Additional file 13: Figure S11. The multiple functions of melanin in a cell and, especially, the resistance to antifungal drugs and survival of the host immune system, are a strong motivation for the study of the genetic characteristics of melanin biosythesis.

## Conclusions

In this study we provide high quality genomic sequence assemblies and annotations for *S. schenckii* and *S. brasiliensis*. Genomic analyses showed a convergent evolutionary fate compared to other dimorphic fungi, even though *Sporothrix* is a close relative of plant-associated Sordariomycetes. Similar to other dimorphic fungal pathogens we have observed a lack of polysaccharide lyase genes which are associated with decay of plants, suggesting evolutionary adaptations from a plant pathogenic or saprobic to an animal pathogenic life style. In addition, convergent branches linking *Sporothrix* and other pathogenic dimorphic fungi were also observed in genes involved in signal transduction and secondary metabolism which suggest similar evolutionary traits. The recent hypothesis of habitat shift from a saprobic life style in fermented plant material to mammal transmission may explain numerous plant/related atavisms. Comparative genomics reveals a certain degree of specialization in the *Sporothrix* lineage which may contribute to our understanding of how fungal-environment-human interactions lead to the selection of pathogenic phenotypes of these species. The *Sporothrix* system may bring new opportunities for functional studies in order to understand the biology of fungi and infection.

## Methods

### Fungal strains and DNA extraction

*Sporothrix schenckii* strain 1099–18 (ATCC MYA-4821) was originally obtained from the Mycology Section, Department of Dermatology, Columbia University, New York, isolated from a patient manifesting subcutaneous sporotrichosis, and has been widely used in experiments of cell wall composition and virulence studies in mice models [97, 106]. *Sporothrix brasiliensis* strain 5110 (ATCC MYA-4823) was isolated from a feline skin lesion in the epidemic area of sporotrichosis in Rio de Janeiro, Brazil, presenting high virulence in mouse model [77]. Mycelial cells were cultivated in Sabouraud broth at 25°C, with shaking (150 rpm) for 14 days, collected by centrifugation and washed 3 times with Phosphate-buffered saline (PBS) solution. Cells were disrupted using the Precellys®24-Dual (Bioamerica) with help of CK28 hard tissue homogenizing tubes. DNA extraction was performed using Qiagen DNeasy Plant Mini Kit, according to manufacturer’s protocols.

### Genome sequencing and assembly

*Sporothrix schenckii* and *S. brasiliensis* genomes were sequenced using next generation 454 pyrosequencing (Roche). Shotgun and paired-end 3 kb inserts libraries were constructed and sequenced in the 454 GS FLX platform according to Roche’s protocols at the Computational Genomics Unity of the National Laboratory for Scientific Computing (LNCC, Petrópolis, RJ, Brazil). Genomic assemblies were carried out using Newbler and Celera Assembler. Sequence gap filling and the removal of contigs corresponding to rDNA genes were manually done, decreasing the numbers of scaffolds and contigs. The assembled scaffolds generated by the two species were aligned and oriented using MAUVE [107]. Similarity scores and dot-plot graphs were generated using LALING/PLALING (http://fasta.bioch.virginia.edu/fasta_www2/fasta_www.cgi?rm=lalign).

### *Ab initio*Gene prediction, annotation and protein family classification

Gene predictions were performed using three different approaches: SNAP [108], AUGUSTUS [109] and EXONERATE [110] using ORF’s identified in the *G. clavigera* strain kw1407/UAMH 11150 [26] as reference and for training and genomic comparisons. Proteins deduced for *G. clavigera* proteome were aligned to the *S. brasiliensis* and *S. schenckii* assembled genomes using Exonerate (percent threshold equal 50) with the model protein2genome. Gene predictions (SNAP and AUGUSTUS) and protein (EXONERATE) alignments were used as input in order to identify consensus gene structures using EVidenceModeler [111]. Consensus ORF’s were subjected to Blast searches against NCBI refseq_protein, KEGG and SwissProt databases. Automatic annotations were performed using SABIA - upgraded for eukaryotic organisms [112] and validated ORF’s were considered with minimum query/subject coverage of 60% and minimum positive 50%. In addition, gene categories according KEGG were inspected manually in order to re-assemble the metabolic pathways of *S. brasiliensis* and *S. schenckii.* Alignments were made by Blastp and the lowest e-value was used to consider homologous sequences. Next, loci identified in *S. schenckii* and *S. brasiliensis* genomes were blasted against the genome libraries of 14 selected fungi (*Neurospora crassa*, *Aspergillus nidulans*, *A. fumigatus*, *Talaromyces marneffei*, *Paracoccidioides lutzii*, *P. brasiliensis*, *Coccidioides immitis*, *Blastomyces dermatitidis*, *Histoplasma capsulatum*, *Fusarium graminearum*, *Magnaporthe oryzae*, *Sordaria macrospora*, *Verticillium dahliae*, *Grosmannia clavigera*) to infer the putative orthologues. Gene products were categorized according to biological process, cellular component and molecular function using GeneOntology (GO) using Blast2GO. Secreted proteins were identified using SignalP3.0 (http://www.cbs.dtu.dk/services/SignalP/) using hidden Markov model.

The prediction of mobile genetic entities was performed by similarity searches using the following approaches and databases: a) Nucleotide Blast against Repbase version 17.10 (http://www.girinst.org/repbase/) [113], Dfam database version 1.1 [114] and Gypsy database version 2.0 (GyDB) [115]; b) PSI-BLAST (Position-Specific Iterated BLAST) using profiles of proteins corresponding to major clades/families of Transposable Elements (TEs) implemented with TransposonPSI tool (http://transposonpsi.sourceforge.net/); c) reverse position-specific BLAST algorithm (RPSBLAST) against Conserved Domain Database (CDD) version March 2013 [116]; and d) tblastn taking specific protein subsets against the *Sporothrix* genome. These subsets were built from NCBI Non Redundant (NR) database version March 2011 using particular description terms related to transposable elements including (apurinic/apyrimidinic endonuclease, aspartic proteinase, ATPase, endonuclease, envelope, GAG protein, helicase, integrase, polymerase B, replication protein A, reverse transcriptase, RNase, transposase, tyrosine transposase/recombinase). In addition, the Tandem repeat finder (TRF) algorithm was used for finding tandem repeats [117]. Transposable elements were classified accordingly [118]. All results were obtained using locally compiled databases. Perl scripts were built for automation of genome scans, report generation and data integration. Artemis sequence visualization and annotation tool [119] was used for manual curation and annotation of transposable elements.

Whole genome gene families were identified using InterproScan combined with Pfam domain assignments. Annotation of carbohydrate-active enzymes was performed in a two-step procedure where the translated protein sequences were compared to the full length sequences derived from the Carbohydrate-Active enZymes (CAZy) database (http://www.cazy.org; [120]) using BLAST [121]. The query sequences that had an e-value <0.1 were subjected to a BLAST search against sequence fragments corresponding to individual catalytic and carbohydrate-binding modules described in CAZy, along with a HMMer search [122] using hidden Markov models corresponding to each CAZy module family. A family assignment was considered reliable when the two methods gave the same result. Borderline cases were resolved by inspection of conserved features such as the presence of known catalytic residues.

### Gene family expansion and contractions

Gene families were determined using OrthoMCL approach comparing with other 13 fungi (Additional file 2: Table S1). Domains were annotated for each orthologous cluster using Interpro [123], Pfam [124] and SMART [125] databases. Significant enrichment or depletion of domains in the *Sporothrix* lineage were calculated based on hypergeometric comparisons (P < 0.05) and the reported p-values are adjusted for multiple testing using q-value [126, 127]. The expanded families with highest discrepancies between *Sporothrix* and other compared fungi were individually analyzed. Protein sequences of LysM, PKS enoyl reductase, Ras, Rab and Rho small gtpases we individually aligned using ClustalW [128] and the domains were manually checked. Uninformative positions of the alignment were eliminated using trimal [129] and the best-fit protein substitution model was inferred based on likelihood values under AIC criteria, implemented in ProtTest [130]. Phylogenetic analysis of expanded gene families were carried out using Maximum likelihood methods implemented in PhyML 3.0 software and 1.000 of non-parametric bootstraps were tested for branch support [131]. Gene duplications/losses for the given family trees were inferred using Notung 2.6 software [132].

### Blast reciprocal best hit and phylogenomic analysis

Bidirectinoal-best Blast Hit (BBH) were performed using two different datasets: first we performed the comparisons between *S. schenckii*, *S. brasiliensis* and *G. clavigera* genomes in order to identify the unique genes in *Sporothrix* lineage. In addition, Blast reciprocal best hits were performed to identify common orthologues in 25 fungal genomes (Additional file 2: Table S1) using minimum query/subject coverage of 50% and e-value of E ≤ 1×10^−20^. A total of 395 orthologs were found in all species analyzed and were aligned using MAFFT [133] and retrieved alignments were trimmed using Trimal [129] in order to exclude spurious sequences or poorly aligned regions. Phylogenomic analysis was performed using RAxML [134] and the Dayhoff aminoacid substitution model was selected according ProtTest [130]. Divergence time between species was calculated with help of r8s v 1.8 [135] program using Langley-Fitch model [136] considering the origin of the Ascomycota at 500 to 650 MYA (Millions Years Ago) [137].

### Cell wall protein/adhesin analysis

Two programs were used for the prediction of cell wall proteins/adhesins: ProFASTA [138] and FungalRV [139]. Analysis using the fasta files of the complete genomes of *S. schenckii* and *S. brasiliensis* were performed for prediction of GPI-anchor secretion signal and transmembrane domain identification. The SignalP 4.0 server (http://www.cbs.dtu.dk/services/SignalP/) was applied for prediction of the presence and location of signal peptide cleavage sites in amino acid sequences, using the method of Input sequences, which do not include TM regions. Then, the TMHMM Server v. 2.0 (http://www.cbs.dtu.dk/services/TMHMM/) was used for prediction of transmembrane helices in proteins, and finally the Big-PI fungal predictor [140] was used for GPI modification sites. The ProFASTA requires the combination of these three analyses to prospect cell wall proteins and adhesins, with the following parameters: SignalIP 4.0 positive; TMHMM 2.0 < 1 helices and number of AA to exclude as 45 from N-terminus and 35 for C-terminus; Big-PI positive. The FungalRV validates only proteins with score up to 0.5 for adhesin or adhesin-like features.

### Autophagy, peroxisome and endocytosis

The initial tool used for this annotation was the KEGG automatic classification, which adequately identified genes involved in peroxisome biogenesis. However, the automatic annotation algorithms only picked up a few genes involved in autophagosome biogenesis and endocytosis, so different approaches were necessary. For the genes involved in autophagy, we started by collecting on the SGD all protein sequences annotated as involved in autophagy and autophagosome biogenesis in *Saccharomyces cerevisiae*. All of these sequences were blasted against the *S. schenckii* and *S. brasiliensis* databases in order to correctly identify homologous protein sequences. To narrow down the list, we focused the analysis on 17 genes that are necessary for autophagosome biogenesis in yeast [141] plus those that are shown on KEGG. Regarding endocytosis, the KEGG table only showed two genes involved in the process itself and several genes involved in vacuolar degradation. To overcome this limitation, the genes that were used for annotation were those listed in a review article as being involved in clathrin-mediated endocytosis in *S. cerevisiae* [142]. All protein sequences encoded by these genes were blasted against the *S. schenckii* and *S. brasiliensis* databases.

### Availability of supporting data

The data sets supporting the results of this article are included within the article and its additional files.

## Electronic supplementary material

Additional file 1: **Core genes for general and secondary metabolism.** (DOCX 56 KB)

Additional file 2: **Table S1.** Information about the 25 genomes retrieved for phylogenomic inference. **Table S2.** Pfam domain expansions/contractions of *Sporothrix schenckii* and *S. brasiliensis*. **Table S3.** SMART domain expansions/contractions of *Sporothrix schenckii* and *S. brasiliensis*. **Table S4.** INTERPRO domain expansions/contractions of *Sporothrix schenckii* and *S. brasiliensis*. **S5.** Comparative genomic analysis of carbohydrate active enzymes (CAZy) of *Sporothrix schenckii* and *S. brasiliensis*. **Table S6.** Putative adhesins of *S. schenckii*. **Table S7.** Putative adhesins of *S. brasiliensis*. **Table S8.** Cell wall and adhesin-related genes characterized in *Sporothrix schenckii* and *S. brasiliensis*. **Table S9.** Glucan synthase and glucanase genes identified in *Sporothrix schenckii* and *S. brasiliensis*. **Table S10.** Chitin synthase and chitinase genes identified in *Sporothrix schenckii* and *S. brasiliensis*. **Table S11.** Genes involved in N-linked glycosylation in *Aspergillus nidulans* and *Neurospora crassa,* and their putative orthologs in *Sporothrix schenckii and S. brasiliensis*. **Table S12.** Enzymes activities related with protein glycosylation in *Aspergillus nidulans* and *Neurospora crassa*, and their putative orthologs in *Sporothrix schenckii and S. brasiliensis*. **Table S13.** Genes involved in O-linked glycosylation in *Aspergillus nidulans* and *Neurospora crassa*, and their putative orthologs in *Sporothrix schenckii*a and *S. brasiliensis*. **Table S14.** Genes involved in GPI-anchor elaboration in *Aspergillus nidulans* and *Neurospora crassa*, and their putative orthologs in *Sporothrix schenckii* and *S. brasiliensis*. **Table S15.** Melanin biosynthesis pathway and putative orthologs in *Sporothrix schenckii* and *S. brasiliensis*. **Table S16.** Comparative analysis of core genes related to amino acids, Secondary, Energy, Cofactor and Vitamin metabolisms of *Sporothrix schenckii* and *S. brasiliensis*. **Table S17.** Genomic identification and classification of phospholipases A, C, and D enzyme families in *Sporothrix schenckii* and *S. brasiliensis* genomes. **Table S18.** Homologs of vitamin and cofactor genes presented analyzed by FUNGIpath in both *Sporothrix* species. **Table S19.** Orthologous genes related to catabolism and transport in *Sporothrix schenckii* and *S. brasiliensis.* (XLS 742 KB)

Additional file 3: Figure S2: Phylogenetic distribution of LysM domains containing proteins in *Sporothrix*. The LysM domains are displayed along the taxa (red bars), chitin binding module type 1 (CB1) (blue bars) plus catalytic sites identified (glycoside hydrolase - GH or Pectin Lyase – PL). Gene paralogous duplications are highlighted by red boxes. (PDF 220 KB)

Additional file 4: Figure S3: Unrooted maximum likelihood tree of Ras Small GTPase proteins (IPR020849) family shows high diversification in the *Sporothrix* lineage. Clades harboring *Sporothrix* and dimorphic fungi are highlighted in red. (PDF 3 MB)

Additional file 5: Figure S4: Unrooted maximum likelihood tree of Rho Small GTPase proteins (IPR003578) family shows high diversification in the *Sporothrix* lineage. Clades harboring *Sporothrix* and dimorphic fungi are highlighted in red. (PDF 2 MB)

Additional file 6: Figure S1: Gene tree and species tree reconciliation of LysM domain-containing genes showing specific *Sporothrix* duplications (blue boxes). (PDF 34 KB)

Additional file 7: Figure S5: Unrooted maximum likelihood tree of Rab Small GTPase proteins (IPR003579) family shows high diversification in the *Sporothrix* lineage. Clades harboring *Sporothrix* and dimorphic fungi are highlighted in red. (PDF 3 MB)

Additional file 8: Figure S6: Gene tree and species tree reconciliation of small GTPase Ras gene family showing independent gene losses in other species and the increased copy number in *Sporothrix* (blue boxes). (PDF 118 KB)

Additional file 9: Figure S7: Gene tree and species tree reconciliation of small GTPase Rho gene family showing independent gene losses in other species and the increased copy number in *Sporothrix* (blue boxes). (PDF 129 KB)

Additional file 10: Figure S8: Gene tree and species tree reconciliation of small GTPase Rab gene family showing independent gene losses in other species and the increased copy number in *Sporothrix* (blue boxes). (PDF 204 KB)

Additional file 11: Figure S9: Chart pies showing the efficiency of the algorithms used to predict the putative adhesins and/or cell wall GPI-anchored proteins of (A) *S. schenckii* (n = 118) and (B) *S. brasiliensis* (n = 106). The relative percentage of putative adhesins and/or GPI- anchored proteins, predicted by either ProFASTA or Fungal RV, is shown as well as the proteins in common by both predictors. (PDF 75 KB)

Additional file 12: Figure S10: Phylogenetic tree of relatedness of *Sporothrix* spp. chitin synthases. The Mega 4 software package was employed, using ClustalW for sequence alignment. Construction of the phylogenetic tree was done by the neighbor-joining method using 1000 replications. The seven chitin synthases identified for both, *Sporothrix brasiliensis* and *S. schenckii*, cluster within the seven chitin synthase classes (I to VII) previously reported [83]. GenBank accession numbers of sequences, and names of fungal species used for construction of the tree are displayed in Additional file 12: Table S10. (TIFF 799 KB)

Additional file 13: Figure S11: Melanin biosynthesis pathways for DHN-Melanin, DOPA-melanin and pyomelanin proposed for *S. schenckii* and *S. brasiliensis* based on melanin biosynthetic pathways described in other pathogenic fungi. The putative enzymes identified in the genomes of *S. schenckii* and *S. brasiliensis* are indicated in circles. Locus tags, Gene products, numbers of exons, size of transcripts, estimated protein sizes and current annotations are listed. (PNG 412 KB)
