# Supplementary material for: Comparative genomics of the major fungal agents of human and animal Sporotrichosis: Sporothrix schenckii and Sporothrix brasiliensis
Source: BMC Genomics. 2014 Oct 29;15:943. doi: 10.1186/1471-2164-15-943 (PMC4226871; doi:10.1186/1471-2164-15-943)
Supplement: Supplementary file 1 — Additional file 1: Core genes for general and secondary metabolism. (DOCX 56 KB) [file 12864_2014_6638_MOESM1_ESM.docx]

**Core genes for general and secondary metabolism**

Core genes involved in general and secondary metabolism, showed high degree of conservation when compared to those present in other Sordariomycetes. Among all, including those involved in energy metabolism (**Table S16**), only two genes (*F-type H+-transporting ATPase subunit C* and *nitrite reductase NAD(P)H large subunit*) were simultaneously present in *S. schenckii* and were not identified in the Eurotiomycetes genomes. The gene encoding subunit C of FoF1-ATPsynthase was present in both *S. schenckii* and *S. brasiliensis* as well as in the reference Sordariomycetes, but it was not conserved in the pathogenic dimorphic fungi. Probably, subunit C is not critical for the assembly and function of the FoF1-ATPsynthase in these dimorphic fungi, because their oxidative phosphorylation chain is functional and capable of oxidative ATP synthesis [[1-4](#_ENREF_1)]. The nitrite reductase gene has also been correlated to the survival of filamentous fungi and persistence in hypoxic environments [[5](#_ENREF_5)]. The nitrite reductase gene was present only in *S. schenckii* and in the class of Sordariomycetes fungi. Intriguingly, *S. brasiliensis* has no gene related to reduction or oxidation of both nitrate and nitrite. Hence, *S. schenckii* could have certain advantages compared to *S. brasiliensis* and other dimorphic fungi regarding environmental survival capabilities.

Genes involved in lipid metabolic pathways in *S. schenckii* and *S. brasiliensis* were identified based on previously annotated Sordariomycetes genomes **(Table S17).** Genome analysis of both species allowed us to identify several genes encoding **phospholipases.** Accordingly, a gene encoding for phospholipase A2 (PL2A) was identified in both *Sporothrix* species. The PLA2 homolog gene of *S. schenckii* was previously reported by [[6](#_ENREF_6)] and is supposed to interact with a G-protein subunit playing a role in signal transduction and fungal pathogenesis. We have found four different phospholipase C (PLC) homologous in *S. schenckii* and *S. brasiliensis*. Several studies have shown that PLC activation plays an important role in intracellular signal transduction by regulating intracellular calcium concentrations. In *Magnaporthe grisea* genes encoding PLC had an important role in growth, morphology, sporulation, appressorium development and pathogenesis [[7](#_ENREF_7), [8](#_ENREF_8)]. Finally, we identified four candidate homologous genes encoding phospholipase D (PLD) isoforms in both *Sporothrix* species which seems to occur in a wide range of fungi belonging to Sordariomycetes. Although the role of PLD in *Sporothrix* is unclear, in *Aspergillus fumigatus* it was shown to regulate conidial internalization into lung epithelial cells. Additionally, a mutant strain for *pld* was less virulent in immunosuppressed mice, suggesting that this gene may encode a virulence factor of *A. fumigatus* [[9](#_ENREF_9)].

We have observed that only three enzymes of amino acid metabolism pathways identified in the genomes of *S.* *schenckii* and *S. brasiliensis* were absent in most of the dimorphic fungi **(Table S16).** The first is a monoamine oxidase enzyme belonging to to the tryptophan, arginine, proline, phenylalanine and tyrosine metabolism pathways. It is a flavoenzyme, widely distributed in fungi, bacteria and mammals, [[10](#_ENREF_10), [11](#_ENREF_11)] and involved in the oxidative deamination of primary amines, using ammonia as nitrogen source [[11](#_ENREF_11), [12](#_ENREF_12)]. In the tyrosine metabolism pathway, the enzyme maleylacetoacetade isomerase is coded by two ORFs in *S. schenckii*. This enzyme is present in dimorphic fungi, but absent in sordariomycete species such as *G.* *clavigera* and *M. grisea*. This enzyme participates in the degradation of phenylalanine to fumarate and acetoacetate [[13](#_ENREF_13)]. The enzyme enolase-phospatase E1, belonging to the cysteine and methionine metabolism pathways, is absent in all dimorphic fungi and present in all Sordariomycetes, suggesting that it is probably involved in situations of environmental stress.

Genes encoding enzymes of vitamins and cofactors metabolism **(Table S18)** were found in both genomes, except for only a single enzyme of lipoic acid metabolism, a homologue of *Escherichia coli* lipoate protein ligase A (LplA). Interestingly, this enzyme appears to be absent in the genome of *S. schenckii* and *S. brasiliensis*, but has been found in all Sordariomycetes and dimorphic fungi analyzed. The formyltetrahydrofolate deformylase is present in both *Sporothrix* species, as well as in Sordariomycetes and other members of the subphylum Pezizomycotina. In contrast, this enzyme is absent from the subphylum Saccharomycotina, from Basidiomycota (except *Ustilago maydis*) and even from dimorphic fungi. This enzyme has been described in bacteria, such as *E. coli*, in which it metabolizes formyl-THF to formate and THF in purine and glycine biosynthesis [[14](#_ENREF_14)]. There are two other genes associated with the metabolism of riboflavin, nicotinate and nicotinamide which are present in both *Sporothrix* species and Sordariomycetes, but absent from all dimorphic fungi analyzed. One of them encodes an acid phosphatase that participates in the riboflavin metabolism pathway, and the other encodes a 5’-nucleotidase, an acid phosphatase that participates in the nicotinate and nicotinamide metabolism. It has been suggested that 5’-nucleotidase could be involved in stress response in *E. coli* [[15](#_ENREF_15)].

**Transport and catabolism**

Autophagy in fungi is essential for survival during starvation, but has also been involved in various processes such as morphogenesis, virulence, survival upon phagocytosis, and metal ion homeostasis. The analysis of the genomes of *S. schenckii* and *S. brasiliensis* supports the observation that these fungi probably have fully functional autophagy, peroxisomes and endocytosis pathways. We have focused on the analysis of 20 genes, which include those shown on the KEGG category of “Regulation of autophagy” and others necessary for autophagosome biogenesis in *S. cerevisiae* [[16](#_ENREF_16)]. Of these 20 genes, 16 were readily identifiable in both *S. schenckii* and *S. brasiliensis* **(Table S19).** Atg4 was only found in *S. schenckii*, whereas Atg10, Atg13 and Atg14 orthologous were not identified in either species. Atg4 is a protease that is involved in cleavage of Atg8, a step that is essential during autophagosome formation. Atg10 is part of an ubiquitin-like conjugation system that is essential in the early steps of autophagosome formation [[17](#_ENREF_17)]. Atg13 is a regulatory subunit of the Atg1 kinase complex, which is involved in autophagosome expansion [[18](#_ENREF_18)]. Atg14 is a subunit of a phosphatidylinositol 3-kinase complex involved in regulating autophagy initiation [[19](#_ENREF_19)]. The category “Peroxisome” includes 19 genes involved in the biogenesis of this organelle. Of these genes, 15 were readily identifiable both in *S. schenckii* and in *S. brasiliensis*. Pex12 was only found in *S. brasiliensis*, while also Pex26, Pmp70 and Pxmp2 were absent from all fungal genomes compared. Pex12 is part of a complex including Pex10 and Pex2 that is found in the peroxisomal membrane and is involved in the translocation of peroxisomal proteins from the cytoplasm to the organelle matrix [[20](#_ENREF_20)]. Both Pex10 and Pex2 were found in the *S. schenckii* genome. Further experimental work is necessary to understand the significance of Atg4 lack in *S. brasiliensis* and Pex12 in *S. schenckii*.

In *S. cerevisiae*, 55 genes are involved in endocytosis [[21](#_ENREF_21)]. Of these, 46 genes were found in both *S. schenckii* and *S. brasiliensis*, whereas App1, Arc18 were only found in *S. schenckii* and Aim3, Aim21, Bsp1, Gts1and Scd5 were not found from either species. Little is known about App1 except that it has phosphatide phosphatase activity and that it interacts with proteins in the endocytic pathway [[22](#_ENREF_22)]. Arc18 is part of the multi-subunit Arp2/3 complex [[23](#_ENREF_23)], of which all other subunits are found in both *Sporothrix* genomes. It is thus hard to find any biological significance to the specific lack of these genes in *S. brasiliensis*. Of interest might be the lack of Gts1 and Scd5 from the *Sporothrix* genomes. Both of these genes can be found in the genome sequences of several fungi in the class Saccharomycotina, including pathogenic *Candida* species. The lack of these two genes in the genomes of *Sporothrix* species indicates that despite the conservation of most of the endocytic machinery in fungi, a few differences do exist, which could be important in understanding *Sporothrix* physiology.

References

1. Felipe MS, Torres FA, Maranhao AQ, Silva-Pereira I, Pocas-Fonseca MJ, Campos EG, Moraes LM, Arraes FB, Carvalho MJ, Andrade RV, et al: **Functional genome of the human pathogenic fungus Paracoccidioides brasiliensis.** *FEMS Immunol Med Microbiol* 2005, **45:**369-381.

2. Felipe MS, Andrade RV, Arraes FB, Nicola AM, Maranhao AQ, Torres FA, Silva-Pereira I, Pocas-Fonseca MJ, Campos EG, Moraes LM, et al: **Transcriptional profiles of the human pathogenic fungus Paracoccidioides brasiliensis in mycelium and yeast cells.** *J Biol Chem* 2005, **280:**24706-24714.

3. Woo PC, Lau SK, Liu B, Cai JJ, Chong KT, Tse H, Kao RY, Chan CM, Chow WN, Yuen KY: **Draft genome sequence of Penicillium marneffei strain PM1.** *Eukaryot Cell* 2011, **10:**1740-1741.

4. Sharpton TJ, Stajich JE, Rounsley SD, Gardner MJ, Wortman JR, Jordar VS, Maiti R, Kodira CD, Neafsey DE, Zeng Q, et al: **Comparative genomic analyses of the human fungal pathogens Coccidioides and their relatives.** *Genome Res* 2009, **19:**1722-1731.

5. Takaya N: **Response to hypoxia, reduction of electron acceptors, and subsequent survival by filamentous fungi.** *Biosci Biotechnol Biochem* 2009, **73:**1-8.

6. Valentin-Berrios S, Gonzalez-Velazquez W, Perez-Sanchez L, Gonzalez-Mendez R, Rodriguez-Del Valle N: **Cytosolic phospholipase A2: a member of the signalling pathway of a new G protein alpha subunit in Sporothrix schenckii.** *BMC Microbiol* 2009, **9:**100.

7. Choi J, Kim KS, Rho HS, Lee YH: **Differential roles of the phospholipase C genes in fungal development and pathogenicity of Magnaporthe oryzae.** *Fungal Genet Biol* 2011, **48:**445-455.

8. Rho HS, Jeon J, Lee YH: **Phospholipase C-mediated calcium signalling is required for fungal development and pathogenicity in Magnaporthe oryzae.** *Mol Plant Pathol* 2009, **10:**337-346.

9. Li X, Gao M, Han X, Tao S, Zheng D, Cheng Y, Yu R, Han G, Schmidt M, Han L: **Disruption of the phospholipase D gene attenuates the virulence of Aspergillus fumigatus.** *Infect Immun* 2012, **80:**429-440.

10. Schilling B, Lerch K: **Amine oxidases from Aspergillus niger: identification of a novel flavin-dependent enzyme.** *Biochim Biophys Acta* 1995, **1243:**529-537.

11. Atkin KE, Reiss R, Koehler V, Bailey KR, Hart S, Turkenburg JP, Turner NJ, Brzozowski AM, Grogan G: **The structure of monoamine oxidase from Aspergillus niger provides a molecular context for improvements in activity obtained by directed evolution.** *J Mol Biol* 2008, **384:**1218-1231.

12. Matsumura K, Hisada H, Obata H, Hata Y, Kawato A, Abe Y, Akita O: **A novel amine oxidase-encoding gene from Aspergillus oryzae.** *J Biosci Bioeng* 2004, **98:**359-365.

13. Edwards R, Dixon DP, Walbot V: **Plant glutathione S-transferases: enzymes with multiple functions in sickness and in health.** *Trends Plant Sci* 2000, **5:**193-198.

14. Nagy PL, McCorkle GM, Zalkin H: **purU, a source of formate for purT-dependent phosphoribosyl-N-formylglycinamide synthesis.** *J Bacteriol* 1993, **175:**7066-7073.

15. Marchler-Bauer A, Lu S, Anderson JB, Chitsaz F, Derbyshire MK, DeWeese-Scott C, Fong JH, Geer LY, Geer RC, Gonzales NR, et al: **CDD: a Conserved Domain Database for the functional annotation of proteins.** *Nucleic Acids Res* 2011, **39:**D225-229.

16. Kirisako T, Ichimura Y, Okada H, Kabeya Y, Mizushima N, Yoshimori T, Ohsumi M, Takao T, Noda T, Ohsumi Y: **The reversible modification regulates the membrane-binding state of Apg8/Aut7 essential for autophagy and the cytoplasm to vacuole targeting pathway.** *J Cell Biol* 2000, **151:**263-276.

17. Shintani T, Mizushima N, Ogawa Y, Matsuura A, Noda T, Ohsumi Y: **Apg10p, a novel protein-conjugating enzyme essential for autophagy in yeast.** *EMBO J* 1999, **18:**5234-5241.

18. Cheong H, Yorimitsu T, Reggiori F, Legakis JE, Wang CW, Klionsky DJ: **Atg17 regulates the magnitude of the autophagic response.** *Mol Biol Cell* 2005, **16:**3438-3453.

19. Kihara A, Noda T, Ishihara N, Ohsumi Y: **Two distinct Vps34 phosphatidylinositol 3-kinase complexes function in autophagy and carboxypeptidase Y sorting in Saccharomyces cerevisiae.** *J Cell Biol* 2001, **152:**519-530.

20. Albertini M, Girzalsky W, Veenhuis M, Kunau WH: **Pex12p of Saccharomyces cerevisiae is a component of a multi-protein complex essential for peroxisomal matrix protein import.** *Eur J Cell Biol* 2001, **80:**257-270.

21. Weinberg J, Drubin DG: **Clathrin-mediated endocytosis in budding yeast.** *Trends Cell Biol* 2012, **22:**1-13.

22. Chae M, Han GS, Carman GM: **The Saccharomyces cerevisiae actin patch protein App1p is a phosphatidate phosphatase enzyme.** *J Biol Chem* 2012, **287:**40186-40196.

23. Winter DC, Choe EY, Li R: **Genetic dissection of the budding yeast Arp2/3 complex: a comparison of the in vivo and structural roles of individual subunits.** *Proc Natl Acad Sci U S A* 1999, **96:**7288-7293.
