# Supplementary figures and images for: Comparative genomics of the major fungal agents of human and animal Sporotrichosis: Sporothrix schenckii and Sporothrix brasiliensis
Source: BMC Genomics. 2014 Oct 29;15:943. doi: 10.1186/1471-2164-15-943 (PMC4226871; doi:10.1186/1471-2164-15-943)

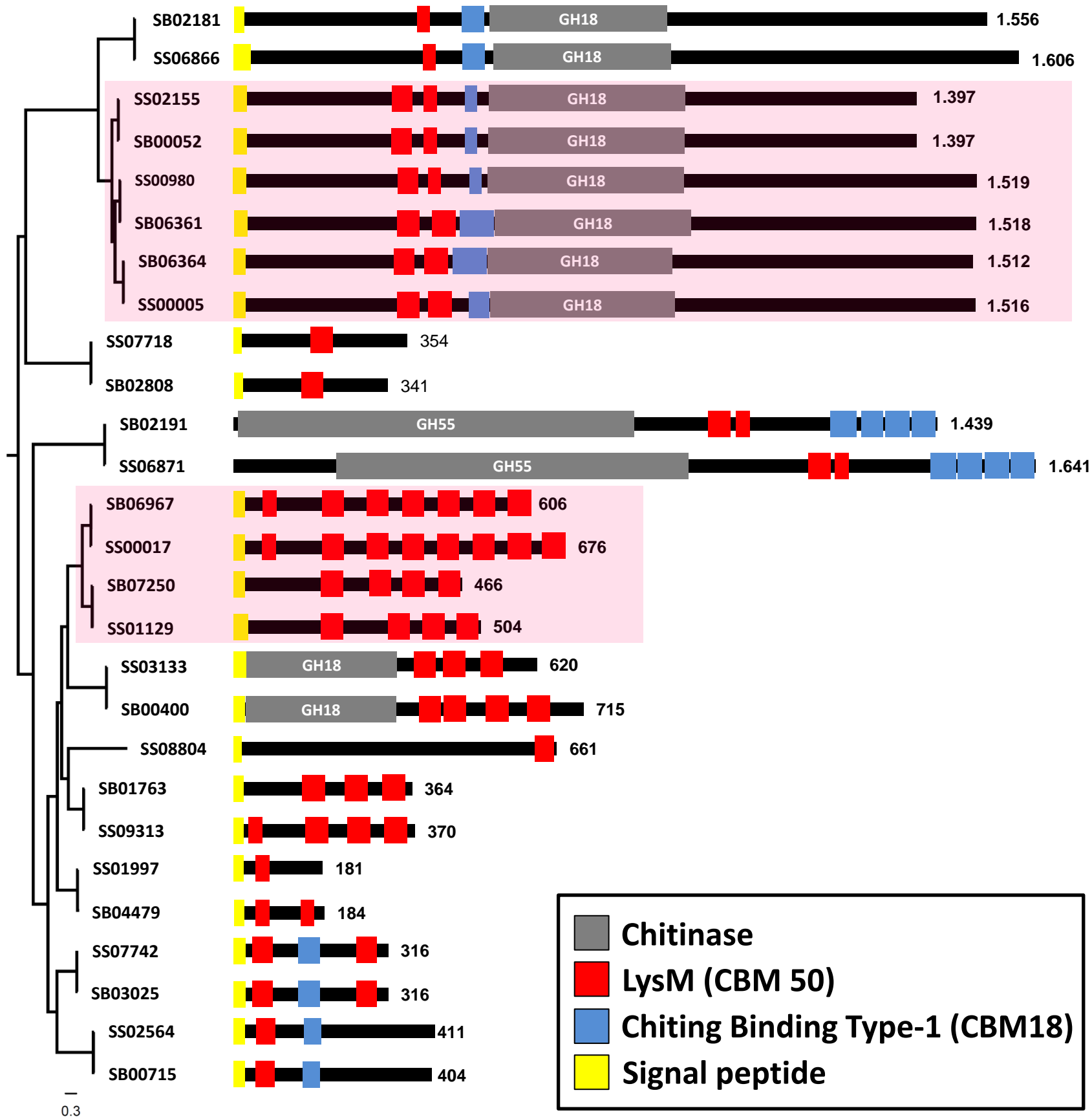

Supplement: Supplementary file 3 — Additional file 3: Figure S2: Phylogenetic distribution of LysM domains containing proteins in Sporothrix. The LysM domains are displayed along the taxa (red bars), chitin binding module type 1 (CB1) (blue bars) plus catalytic sites identified (glycoside hydrolase - GH or Pectin Lyase – PL). Gene paralogous duplications are highlighted by red boxes. (PDF 220 KB) [file 12864_2014_6638_MOESM3_ESM.pdf]

Ras GTPase  
ML – 1.000 bootstraps  
JTT + G

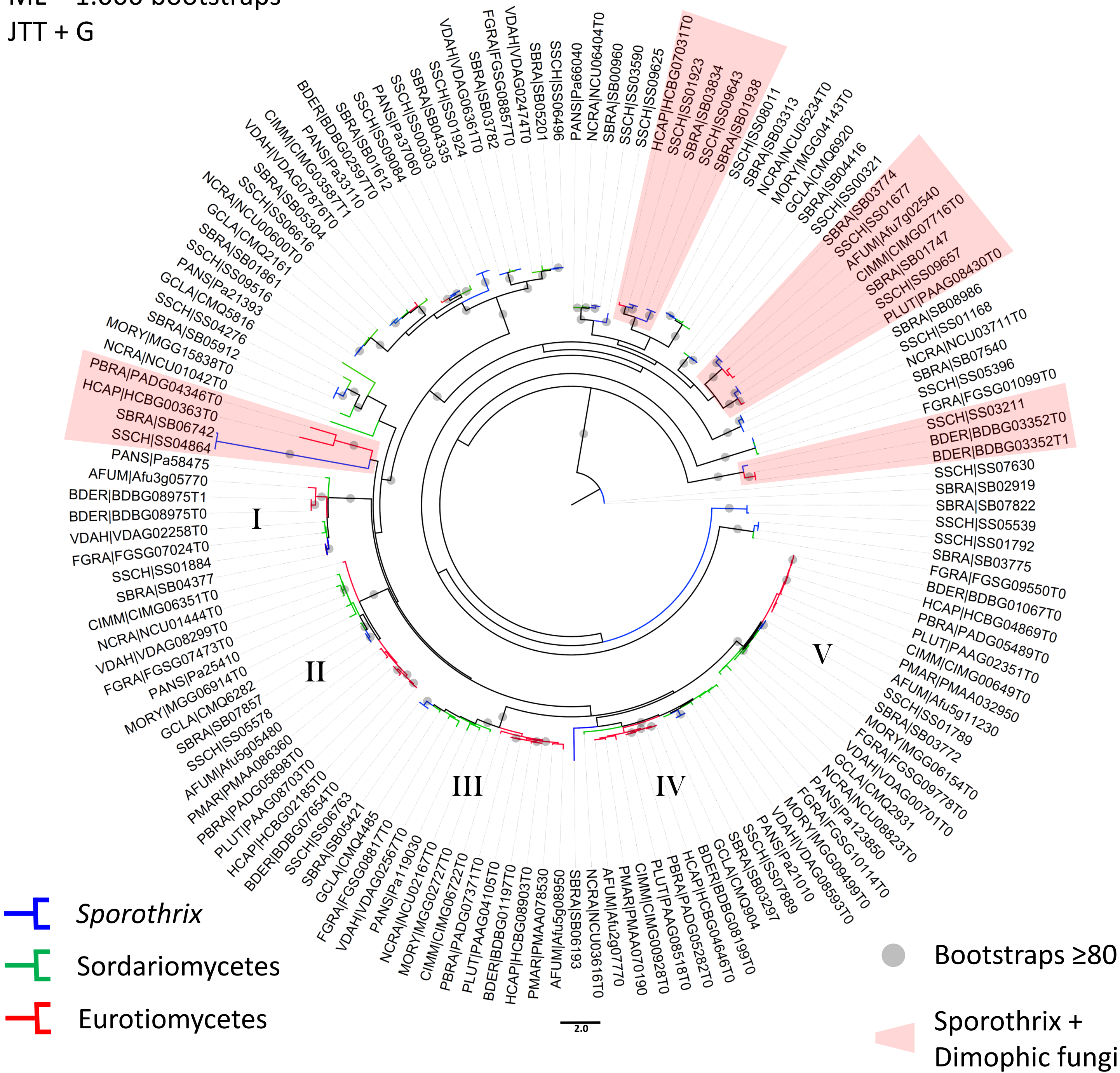

Supplement: Supplementary file 4 — Additional file 4: Figure S3: Unrooted maximum likelihood tree of Ras Small GTPase proteins (IPR020849) family shows high diversification in the Sporothrix lineage. Clades harboring Sporothrix and dimorphic fungi are highlighted in red. (PDF 3 MB) [file 12864_2014_6638_MOESM4_ESM.pdf]

Rho GTPase  
ML – 1.000 bootstraps  
LG + G

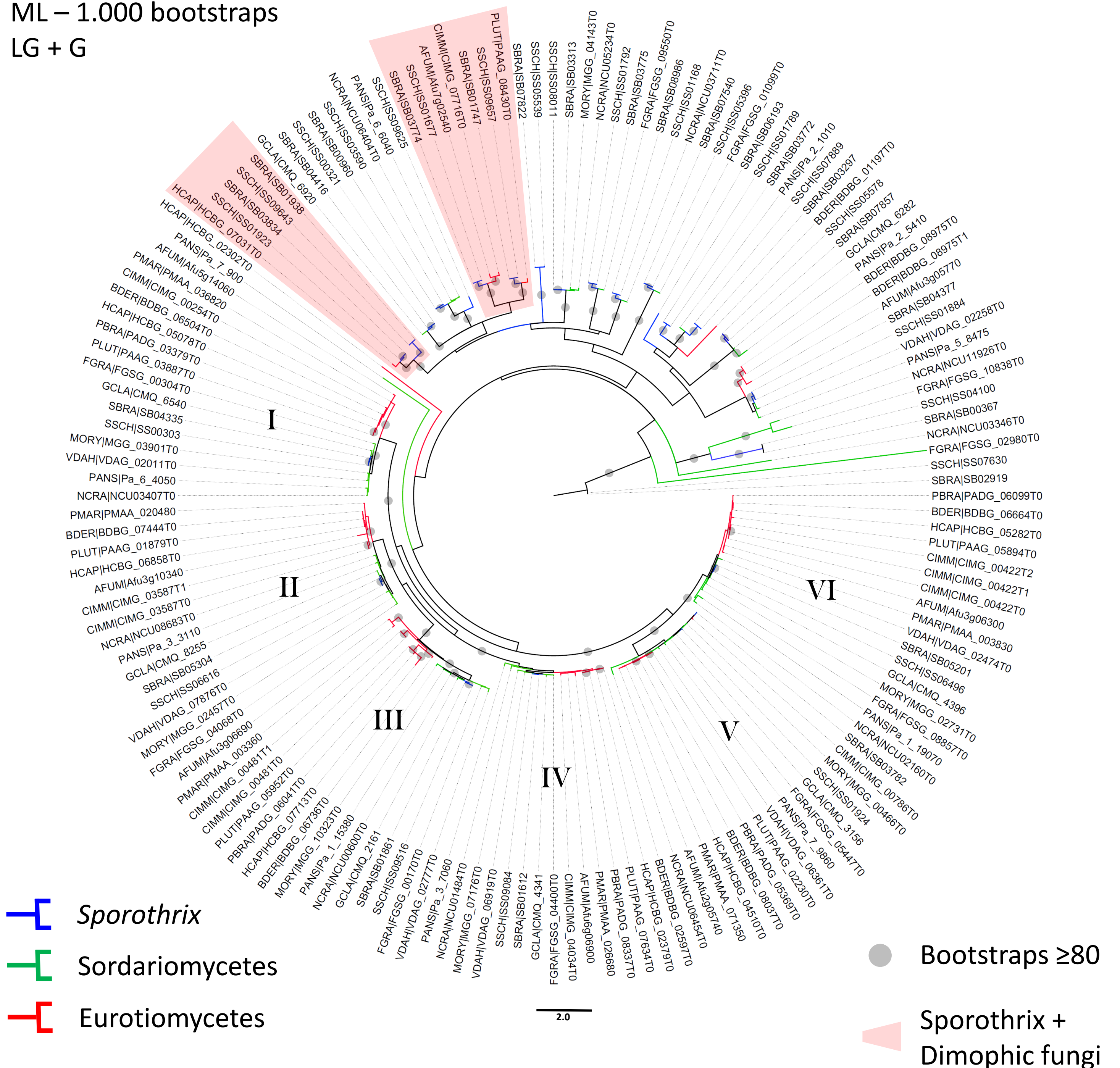

Supplement: Supplementary file 5 — Additional file 5: Figure S4: Unrooted maximum likelihood tree of Rho Small GTPase proteins (IPR003578) family shows high diversification in the Sporothrix lineage. Clades harboring Sporothrix and dimorphic fungi are highlighted in red. (PDF 2 MB) [file 12864_2014_6638_MOESM5_ESM.pdf]

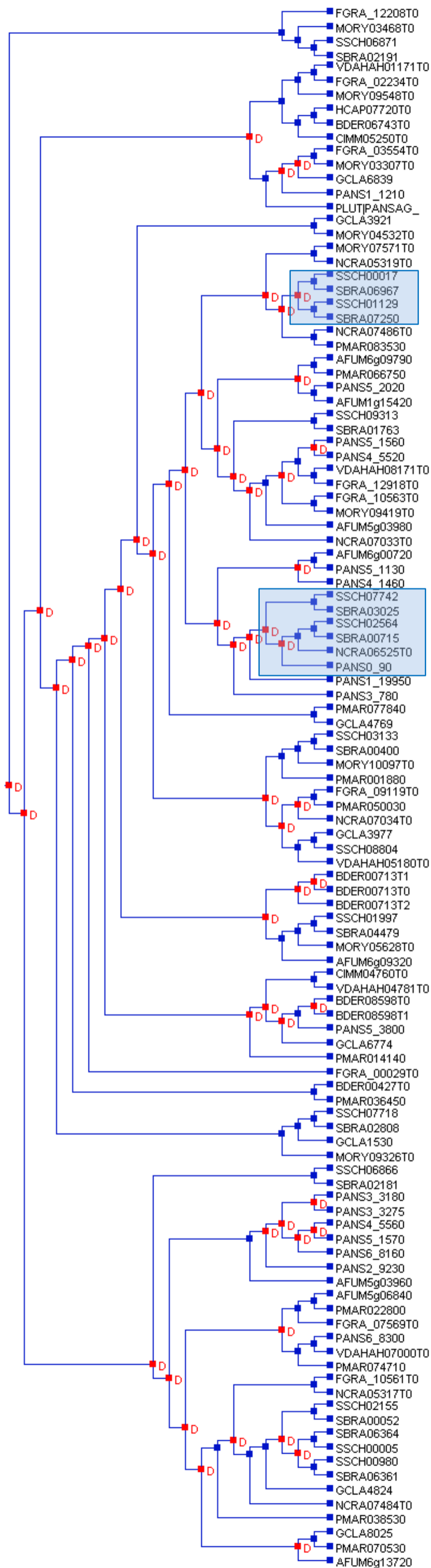

Supplement: Supplementary file 6 — Additional file 6: Figure S1: Gene tree and species tree reconciliation of LysM domain-containing genes showing specific Sporothrix duplications (blue boxes). (PDF 34 KB) [file 12864_2014_6638_MOESM6_ESM.pdf]

Rab GTPase  
ML – 1.000 bootstraps  
LG + G

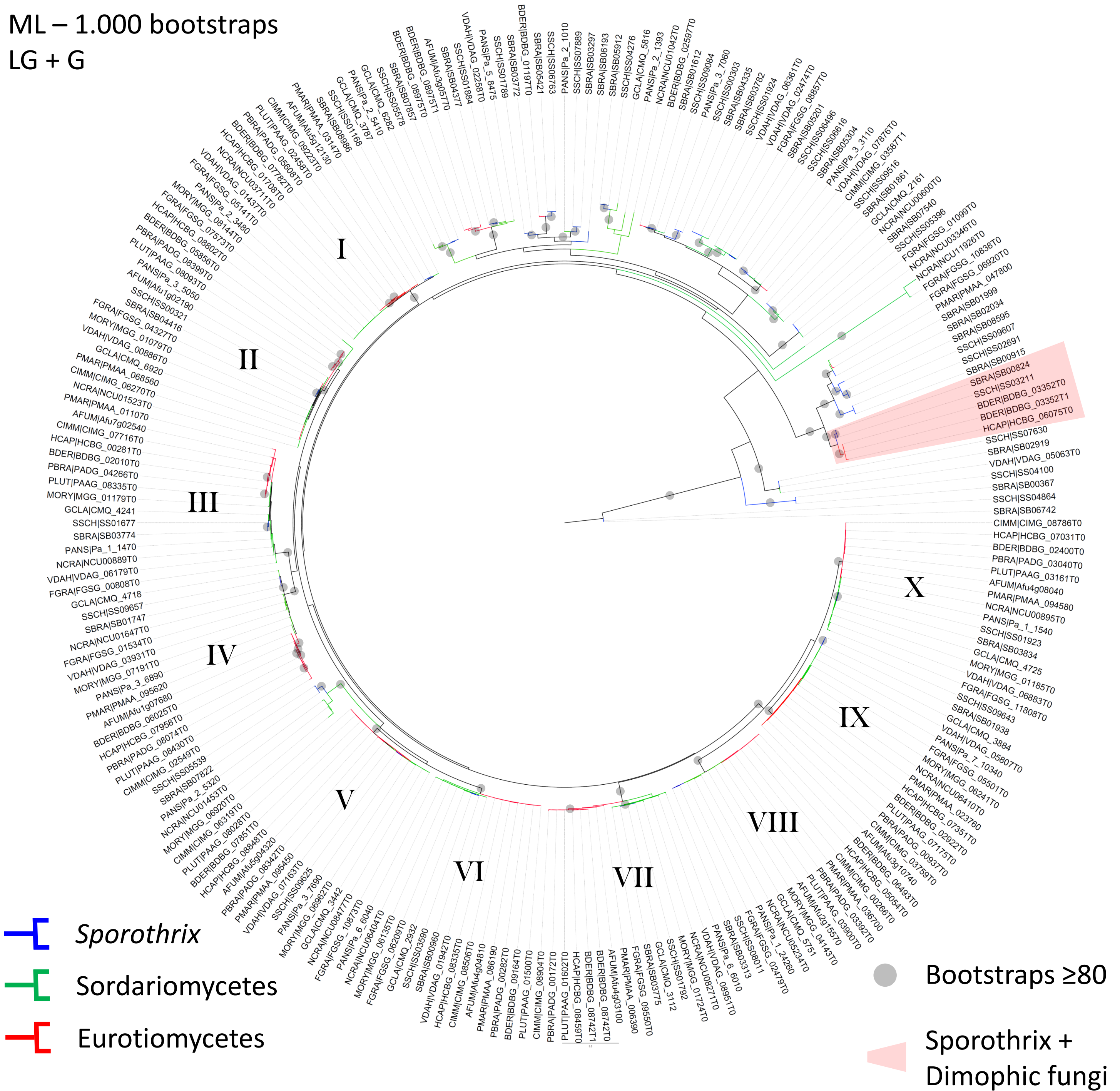

Supplement: Supplementary file 7 — Additional file 7: Figure S5: Unrooted maximum likelihood tree of Rab Small GTPase proteins (IPR003579) family shows high diversification in the Sporothrix lineage. Clades harboring Sporothrix and dimorphic fungi are highlighted in red. (PDF 3 MB) [file 12864_2014_6638_MOESM7_ESM.pdf]

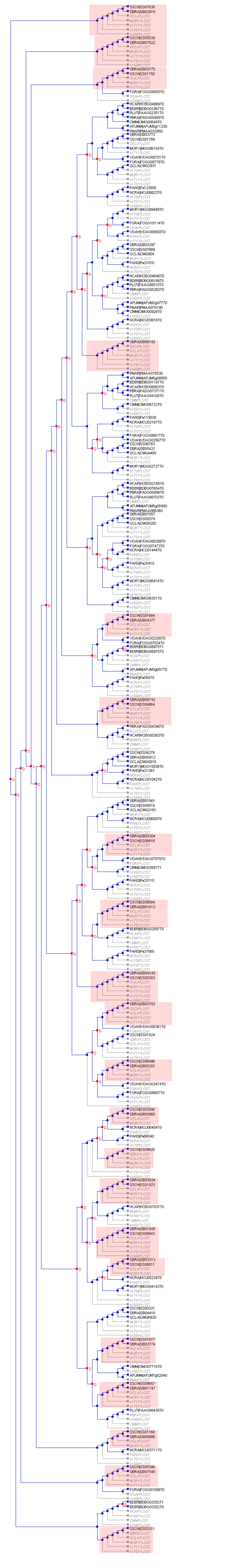

Supplement: Supplementary file 8 — Additional file 8: Figure S6: Gene tree and species tree reconciliation of small GTPase Ras gene family showing independent gene losses in other species and the increased copy number in Sporothrix (blue boxes). (PDF 118 KB) [file 12864_2014_6638_MOESM8_ESM.pdf]

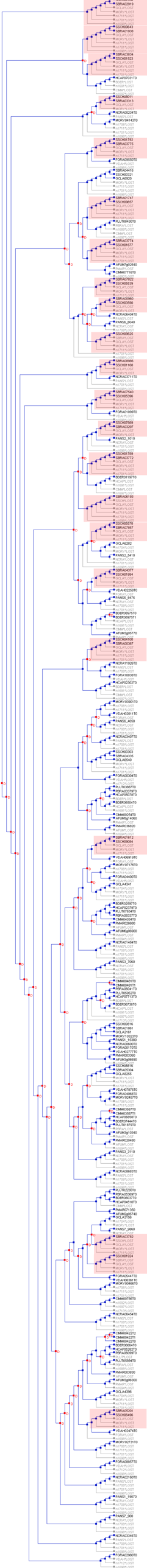

Supplement: Supplementary file 9 — Additional file 9: Figure S7: Gene tree and species tree reconciliation of small GTPase Rho gene family showing independent gene losses in other species and the increased copy number in Sporothrix (blue boxes). (PDF 129 KB) [file 12864_2014_6638_MOESM9_ESM.pdf]

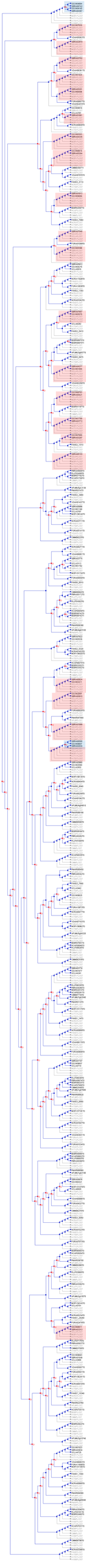

Supplement: Supplementary file 10 — Additional file 10: Figure S8: Gene tree and species tree reconciliation of small GTPase Rab gene family showing independent gene losses in other species and the increased copy number in Sporothrix (blue boxes). (PDF 204 KB) [file 12864_2014_6638_MOESM10_ESM.pdf]

A

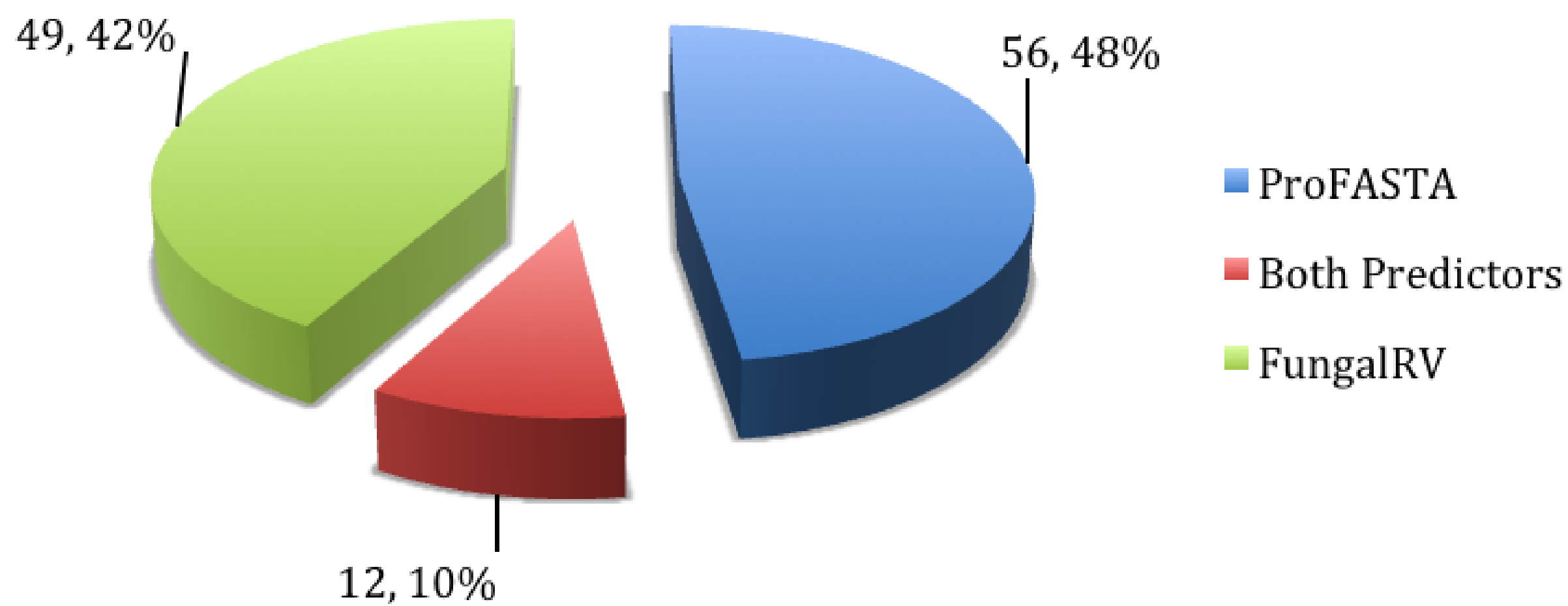

B

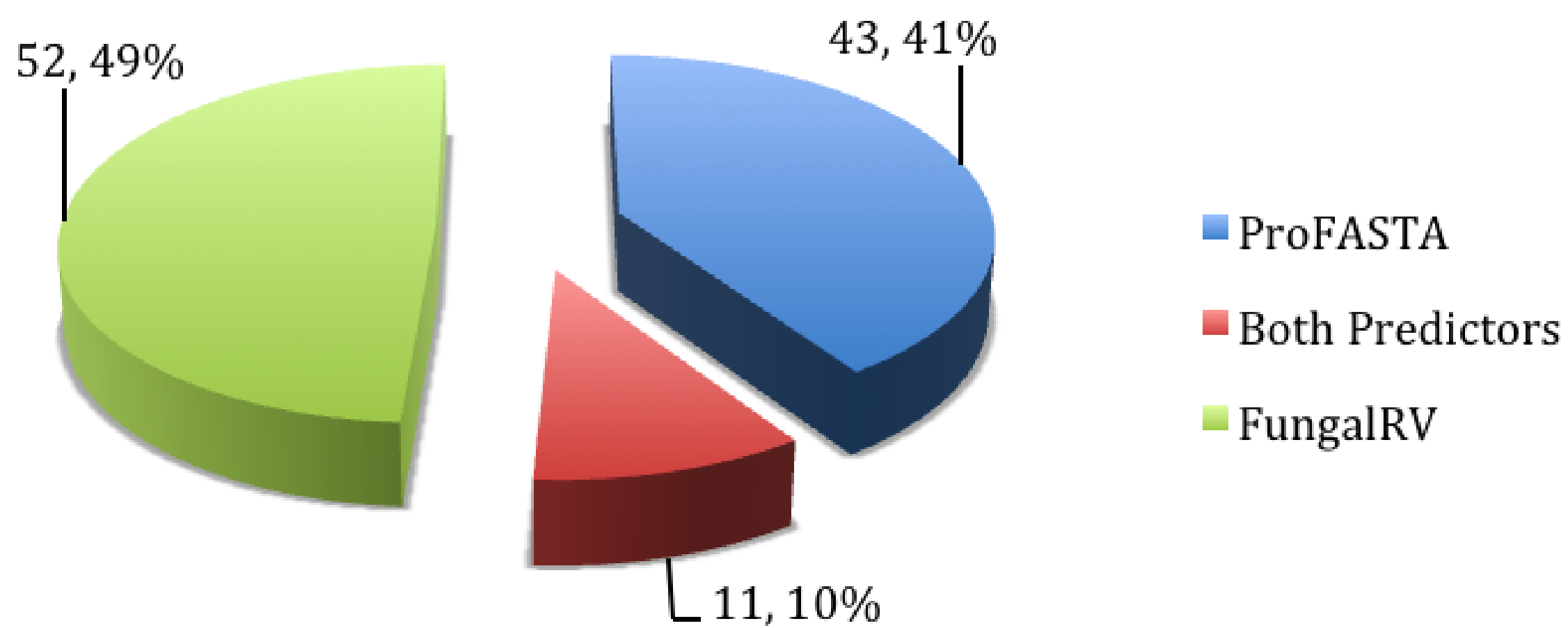

Supplement: Supplementary file 11 — Additional file 11: Figure S9: Chart pies showing the efficiency of the algorithms used to predict the putative adhesins and/or cell wall GPI-anchored proteins of (A) S. schenckii (n = 118) and (B) S. brasiliensis (n = 106). The relative percentage of putative adhesins and/or GPI- anchored proteins, predicted by either ProFASTA or Fungal RV, is shown as well as the proteins in common by both predictors. (PDF 75 KB) [file 12864_2014_6638_MOESM11_ESM.pdf]

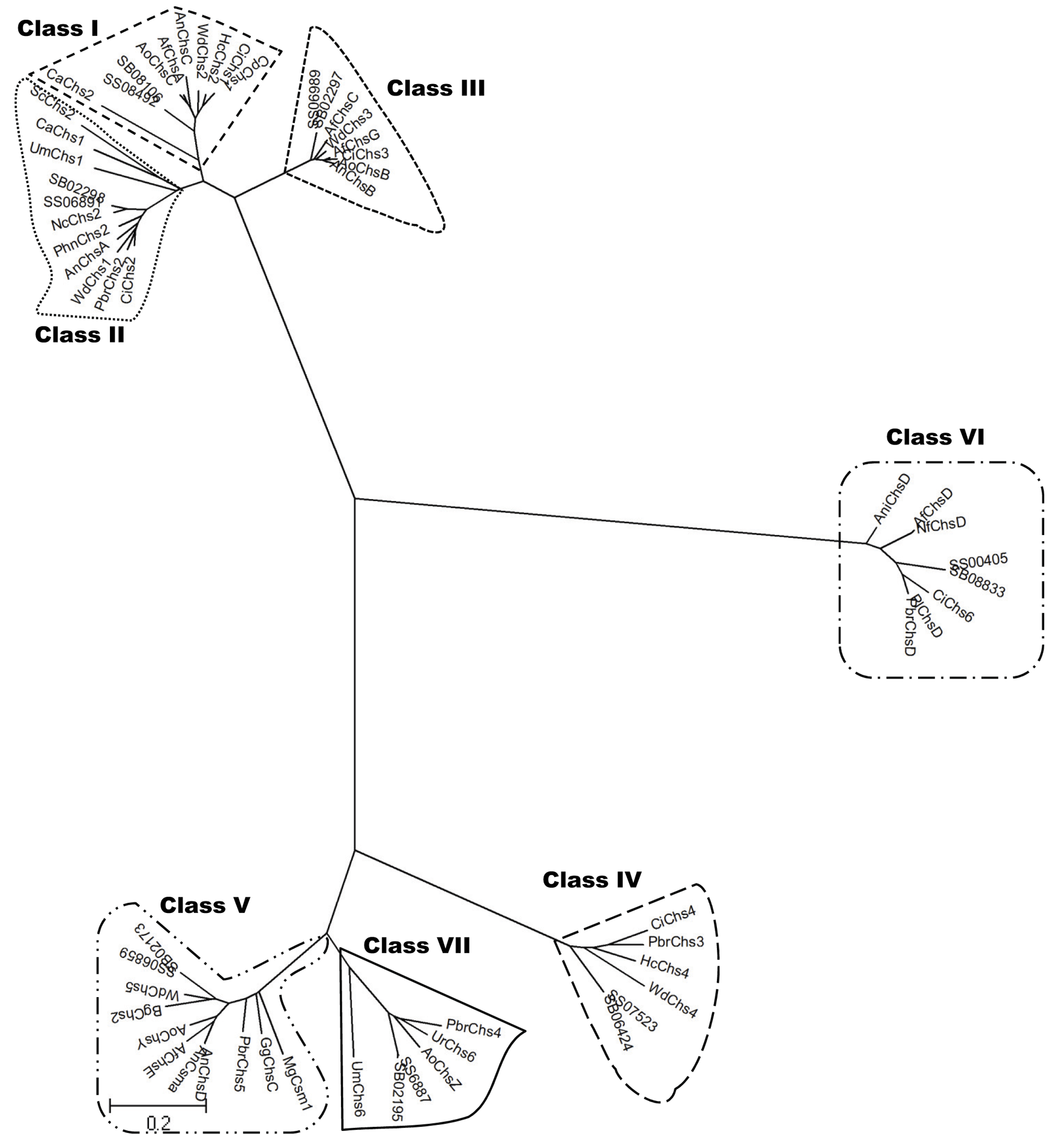

Supplement: Supplementary file 12 — Additional file 12: Figure S10: Phylogenetic tree of relatedness of Sporothrix spp. chitin synthases. The Mega 4 software package was employed, using ClustalW for sequence alignment. Construction of the phylogenetic tree was done by the neighbor-joining method using 1000 replications. The seven chitin synthases identified for both, Sporothrix brasiliensis and S. schenckii, cluster within the seven chitin synthase classes (I to VII) previously reported [83]. GenBank accession numbers of sequences, and names of fungal species used for construction of the tree are displayed in Additional file 12: Table S10. (TIFF 799 KB) [file 12864_2014_6638_MOESM12_ESM.tiff]

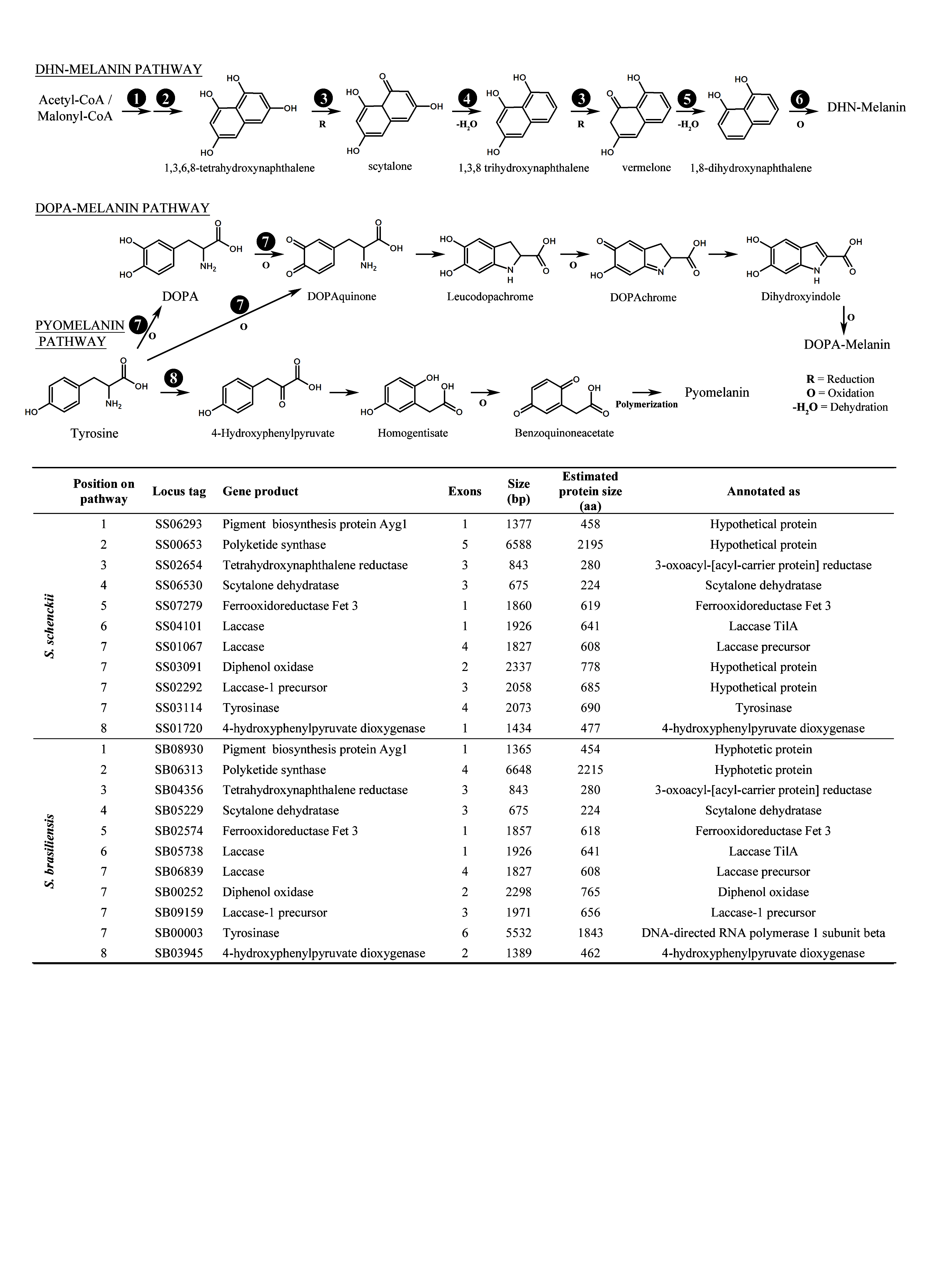

Supplement: Supplementary file 13 — Additional file 13: Figure S11: Melanin biosynthesis pathways for DHN-Melanin, DOPA-melanin and pyomelanin proposed for S. schenckii and S. brasiliensis based on melanin biosynthetic pathways described in other pathogenic fungi. The putative enzymes identified in the genomes of S. schenckii and S. brasiliensis are indicated in circles. Locus tags, Gene products, numbers of exons, size of transcripts, estimated protein sizes and current annotations are listed. (PNG 412 KB) [file 12864_2014_6638_MOESM13_ESM.png]
